# Precision Orthobiologics for Chronic Musculoskeletal Pain: Matching Biology, Pathology, and Patient Phenotype—A Narrative Review of Current Evidence and Future Directions in Regenerative Pain Medicine

**DOI:** 10.3390/medicina62101881

**Published:** 2026-09-28

**Authors:** Giuliano Lo Bianco, Douglas P. Beall, Sudhir Diwan, Sandra M. Martinez, Emanuele Piraccini, Giulia Biondi, Carlo Francesco Biundo, Alaa Abd-Elsayed, Philippe Mavrocordatos, Manuela Baronio, Annu Navani

**Affiliations:** 1 Anesthesiology and Pain Department, Fondazione Istituto G. Giglio Cefalù, 90015 Palermo, Italy; 2 Comprehensive Specialty Care, Oklahoma City, OK 73112, USA; 3 Advanced Spine on Park Avenue, New York, NY 10022, USA; 4 Pain Medicine, Anesthesiology, Llanogrande Clinic, Medellín 050024, Colombia; 5 Unit of Pain Management, Emergency Department, AUSL Bologna, Bellaria Hospital, 40033 Bologna, Italy; 6 Pain Unit, ASST Lariana, 22100 Como, Italy; 7 Department of Anesthesiology, School of Medicine and Public Health, University of Wisconsin, Madison, WI 53792, USA; 8 Swiss Pain Institute, CH-1003 Lausanne, Switzerland; 9 Department of Anesthesia and Critical Care, Azienda Ospedaliero-Universitaria Careggi, University of Florence, 50134 Florence, Italy; 10 Le Rêve Regenerative Wellness, San Jose, CA 95124, USA

**Keywords:** platelet-rich plasma, orthobiologics, precision medicine, osteoarthritis, tendinopathy, discogenic pain, extracellular vesicles, patient phenotyping, prognostic and predictive biomarkers, regenerative pain medicine

## Abstract

*Background and Objectives*: Orthobiologic injectables—principally platelet-rich plasma (PRP), bone marrow aspirate concentrate (BMAC), cell-based therapies and, increasingly, extracellular vesicles—are widely used for chronic musculoskeletal (MSK) pain, yet their evidence base is inconsistent and guideline endorsement is heterogeneous. One frequently proposed contributor to this inconsistency is the use of biologically dissimilar products as interchangeable, one-size-fits-all injections in poorly stratified populations. This narrative review examines current evidence through a conceptual “precision” framework organized around three axes—the biology of the product, the pathology of the target tissue, and the phenotype of the patient—and around image-guided delivery as the procedural context in which these axes are operationalized. It spans osteoarthritis, tendinopathy, plantar fasciopathy, discogenic and spinal pain, and impaired bone healing, with emphasis on PRP, BMAC, and platelet- and cell-derived extracellular vesicles. *Materials and Methods*: PubMed/MEDLINE, Embase, Cochrane CENTRAL and Web of Science were searched from inception to 15 January 2026 and updated on 31 August 2026; 4812 de-duplicated records were screened by two authors independently, and 435 full texts were assessed, from which 76 sources are cited (36 clinical studies and evidence syntheses, 11 guidelines, consensus statements or reporting standards, and 29 narrative, mechanistic, regulatory or methodological references). Evidence was prioritized as guidelines and formal consensus, then syntheses with a certainty assessment, then individual trials, with neutral and negative trials given equal weight. This is a narrative, not a systematic, review; SANRA was used as a critical-appraisal checklist, not as a reporting guideline. *Results*: Across indications, pooled efficacy signals are frequently positive but of low-to-moderate certainty, with substantial risk of bias, small-study effects, and inconsistent product reporting; adequately controlled trials in knee osteoarthritis, Achilles and lateral-elbow tendinopathy, and discogenic pain have returned neutral results. Candidate response modifiers—structural phenotype, inflammatory and metabolic status, pain-processing phenotype, intraprocedural signals, and machine-learning models—are at present largely prognostic associations rather than validated treatment-effect modifiers. Guideline positions diverge, reflecting differences in methodology and evidence thresholds as well as in the underlying evidence. *Conclusions*: We present the three-axis framework as a testable hypothesis and a structure for stratified prospective trials, not as a validated clinical algorithm. We classify its components by clinical readiness, distinguish prognostic from predictive biomarkers, temper regenerative language where only symptomatic benefit is demonstrated, and identify platelet- and cell-derived extracellular vesicles as an early-stage, predominantly preclinical direction whose translational promise remains to be established.

## 1. Introduction

Musculoskeletal conditions are collectively among the largest contributors to years lived with disability worldwide, and low back pain alone is the single leading cause [1]. The principal drivers of chronic musculoskeletal (MSK) pain—osteoarthritis (OA), tendinopathy, plantar fasciopathy, degenerative spinal disease, and impaired bone repair—share a common therapeutic predicament: conventional treatments frequently reduce symptoms without consistently restoring the structure, biological function, or intrinsic healing capacity of the affected tissue. Between the failure of conservative measures and the irreversibility of surgery lies a widening treatment gap that orthobiologic injectables have been widely adopted to fill. Platelet-rich plasma (PRP), bone marrow aspirate concentrate (BMAC), autologous protein solution, cell-based therapies and, most recently, platelet- and cell-derived extracellular vesicles are now used across virtually every MSK indication, frequently ahead of the evidence that would justify their routine adoption [2,3].

The pathobiology that these products are intended to address is itself complex. OA is no longer regarded as a passive “wear-and-tear” process but as a whole-joint disease in which cartilage degradation, synovial inflammation, subchondral bone remodeling, altered chondrocyte and synoviocyte metabolism, oxidative stress, and disturbed micronutrient and trace-element homeostasis interact with mechanical load and systemic factors such as obesity and metabolic disease [4]. This multifactorial biology helps to explain why single-agent interventions—including biologically active injectables—produce variable results, and why the same radiographic grade can conceal very different disease processes. Against this background, PRP has become the most widely used orthobiologic. Meta-analyses generally report advantages over hyaluronic acid (HA) or corticosteroid in knee OA [5,6], but the evidence is characterized by heterogeneous preparations, variable comparators, short follow-up, and a high risk of bias, and adequately powered placebo-controlled trials have returned neutral results [7]. Even in surgical augmentation, a small randomized study of PRP added to arthroscopic microfracture found no significant between-group difference in WOMAC or Lysholm scores at 24 weeks [8]. Whether the residual clinical value of PRP is large enough to matter is exactly what remains unsettled, and it is the question this review is organized around: not “does PRP work?” but “which preparation, for which pathology, in which patient, and delivered how?”.

The promise of orthobiologics is biologically coherent: they deliver a concentrated, autologous or allogeneic payload of growth factors, cytokines, and reparative signaling intended to shift a degenerative or stalled-healing microenvironment toward repair [9,10]. The inconsistency of clinical outcomes is, however, over-determined. Meta-analyses of PRP in knee OA routinely report statistical heterogeneity (I^2^) exceeding 90% [5]. Part of that heterogeneity plausibly reflects the pooling of biologically different products and of patient populations defined only by shared radiographic findings. But heterogeneity also arises from small-study effects, inadequate blinding and allocation concealment, contextual and placebo responses to invasive procedures, selective reporting, inconsistent comparators, differing rehabilitation co-interventions, regression to the mean, and non-equivalent outcome instruments and follow-up windows. Any framework that attributes inconsistency primarily to product–patient mismatch must therefore be treated as a hypothesis to be tested rather than as an established explanation. That is the position adopted throughout this review.

A precision-medicine paradigm offers one way to organize these questions. As articulated in a recent review by Navani et al., precision orthobiologics seeks to move beyond diagnosis-driven prescribing toward biologically tailored intervention, with patient stratification informed by molecular, inflammatory, genetic, and multi-omic markers [2]. That review is a direct precedent for the present work, and the relationship between the two is set out explicitly in Table 1. Our contribution is deliberately complementary: it broadens the clinical scope from the joint to the full span of chronic MSK pain seen in interventional practice; it adds an explicit procedural axis (image-guided delivery and other procedural variables); it integrates the 2024–2026 evidence, including head-to-head safety data [11], adequately controlled neutral trials, and first-in-human vesicle studies; and, in response to the limited maturity of the field, it classifies every component of the framework by clinical readiness rather than presenting it as a unified recommendation. We organize the review around a simple, testable thesis: that the probability of a clinically meaningful response to an orthobiologic depends jointly on the biology of the product, the pathology of the target tissue, and the phenotype of the patient. Where any one axis is unmeasured, outcome variability is expected to increase. Whether aligning the three axes actually improves outcomes relative to conventional indication-based treatment is an empirical question that no prospective trial has yet answered.

This framing is directly relevant to interventional pain medicine, where orthobiologics are delivered not as systemic drugs but as precisely placed injections, and where the guiding principle is increasingly to match the intervention to the underlying etiology rather than to the symptom alone [12]. In that setting the procedural context of the three axes becomes explicit: accurate targeting of the symptomatic lesion under ultrasound or fluoroscopic guidance, appropriate needle placement (intra-articular, peritendinous, intradiscal, or subchondral), and confirmation that the biologic reaches the intended compartment. Image guidance demonstrably improves the accuracy of injection into several joints and peri-tendinous structures and, for some shoulder targets, short-term efficacy [13,14]; whether it improves outcomes of orthobiologic injection specifically has not been tested in comparative trials, and other procedural variables—injection volume, number and interval of injections, activation, use of local anesthetic, peri-procedural NSAID exposure, and post-injection rehabilitation—may contribute at least as much to heterogeneity (Section 8.3). We therefore treat image-guided delivery as the procedural context of the framework and as a source of variance to be reported, situating regenerative orthobiologics within the broader trajectory of precision interventional pain medicine that also encompasses image-guided neuromodulatory techniques [15,16].

**Table 1:** Scope of the present review compared with the 2025 precision-orthobiologics review by Navani et al. [2].

Dimension	Navani et al. 2025 [2]	Present Review
Primary question	How molecular, imaging, multi-omic and AI tools can individualize orthobiologic therapy	Whether and how product biology, tissue pathology, patient phenotype and procedural delivery jointly explain—and could be used to reduce—outcome heterogeneity [2,17]
Clinical scope	Musculoskeletal regenerative medicine, with emphasis on joint disease	Knee OA, tendinopathy (site-specific), plantar fasciopathy, discogenic/spinal pain, delayed/non-union of long bones, with wound healing as a host-factor model [5,18,19,20,21,22]
Product focus	Broad orthobiologic landscape; delivery systems (hydrogels, scaffolds, nanoparticles)	PRP (LP/LR; classification schemes), BMAC vs. expanded MSCs, autologous protein solution, perinatal products, platelet- and MSC-derived extracellular vesicles [23,24,25,26]
Procedural axis	Not addressed as a separate dimension	Explicit: image guidance, target compartment, injection variables, intraprocedural signals [13,14]
Evidence window	January 2015–December 2024	Emphasis on 2020–2026; includes RESTORE, DREAM, ESSKA-ORBIT Parts 1–2, ESSKA safety meta-analysis, ISTH SSC guidance, MISEV2023, first-in-human exosome trial [7,11,20,23,26,27,28,29]
Critical appraisal	Narrative	Explicit certainty/risk-of-bias appraisal per indication (Section 4); prognostic-vs-predictive distinction; neutral trials given equal weight [30,31]
Guidelines and regulation	Summarized	Guideline positions verified against primary documents (Section 6); FDA/EU pathways cited to primary regulation (Section 9) [32,33,34,35,36,37]
Output	Conceptual paradigm	Three-axis framework presented as a testable hypothesis, with each input classified by readiness (Section 8) and a specific stratified-trial agenda (Section 11) [38,39]

## 2. Scope and Approach

This is a narrative, not a systematic, review. SANRA, the Scale for the Assessment of Narrative Review Articles, is a critical-appraisal instrument for readers and editors rather than a reporting guideline, and completing it does not by itself make a review reproducible; we used it as a checklist against which to audit this manuscript, and the completed assessment is provided as Supplementary Table S1 [40]. Because narrative reviews are vulnerable to selective citation, we set out the search, screening, and selection process in detail below and in Table 2 so that readers can judge coverage and potential selection bias.

### 2.1. Information Sources and Search Strategy

PubMed/MEDLINE, Embase, the Cochrane Central Register of Controlled Trials (CENTRAL), and Web of Science Core Collection were searched from database inception. The initial search was run on 15 January 2026 and updated on 31 August 2026 to capture literature published up to the resubmission date; several 2026 publications cited here were identified in the update. Supplementary sources were the guideline repositories of the ACR/Arthritis Foundation, OARSI, AAOS, VA/DoD, and ESSKA; the reference lists of included reviews; and the regulatory websites of the U.S. Food and Drug Administration (FDA), the European Medicines Agency (EMA), and EUR-Lex. Searches combined controlled vocabulary (MeSH/Emtree) and free-text terms for the product domain (platelet-rich plasma; PRP; platelet concentrate; bone marrow aspirate concentrate; BMAC; mesenchymal stem/stromal cell; orthobiologic*; extracellular vesicle*; exosome*), the indication domain (osteoarthritis; tendinopathy; epicondylitis; rotator cuff; Achilles; plantar fasciitis/fasciopathy; discogenic; intervertebral disc degeneration; low back pain; nonunion; delayed union; diabetic foot ulcer), and the precision domain (precision medicine; personalized; phenotype; biomarker; predictor; responder; machine learning; classification). Table 2 reproduces the core PubMed string in abbreviated form; the complete strings as run in each database, together with the eligibility criteria in full, are provided in Supplementary Table S2 (submitted as a separate file). No language restriction was applied at the search stage; non-English records without an English abstract were excluded at screening.

### 2.2. Eligibility Criteria and Study Selection

Eligible sources were (i) randomized controlled trials (RCTs) and controlled cohort studies of orthobiologic injectables in the target indications; (ii) systematic reviews, meta-analyses, and network meta-analyses of such trials; (iii) mechanistic and product-characterization studies bearing on composition, classification, or biological activity; (iv) clinical practice guidelines and formal consensus documents from professional societies; (v) primary regulatory documents; and (vi) studies reporting predictors or correlates of response. Case reports, conference abstracts, non-peer-reviewed sources, and animal studies were excluded, except that preclinical work was cited where no human data existed (extracellular vesicles) and was labelled as such. Where several systematic reviews addressed the same question, the most recent review with the largest included-trial base and a formal certainty assessment (GRADE or equivalent) was preferred; the others were retained only if they contributed unique data (for example, a network meta-analysis or a safety-specific synthesis). Records were de-duplicated in EndNote. Titles and abstracts were screened by two authors independently (G.L.B. and E.P.); full texts were assessed by the same two authors, with disagreements resolved by discussion with a third author (D.P.B.). Data relevant to design, population, product characterization, comparator, follow-up, effect size, and risk-of-bias assessment were extracted into a structured template by one author and checked by a second.

### 2.3. Prioritization and Appraisal

The combined searches identified 5967 records (5904 from databases and 63 from supplementary sources), leaving 4812 after de-duplication; 4377 were excluded at title/abstract screening, and 435 full texts were assessed, of which 76 are cited in this review. Those 76 sources are not 76 included studies in the sense of a systematic review: they comprise 8 randomized controlled trials, 15 systematic reviews, meta-analyses or network meta-analyses, 13 observational, epidemiological or comparative clinical studies, 11 clinical practice guidelines, consensus statements or reporting standards, 18 narrative or mechanistic reviews and preclinical mechanistic studies, and 11 regulatory or methodological sources (8 + 15 + 13 + 11 + 18 + 11 = 76). The three groups used in the Abstract are aggregates of these six categories: clinical studies and evidence syntheses (8 + 15 + 13 = 36); guidelines, consensus statements and reporting standards (11); and narrative or mechanistic reviews together with regulatory and methodological sources (18 + 11 = 29). The remaining full texts were excluded as superseded, duplicative, or outside scope; a flow summary with the reasons for exclusion is provided in Supplementary Figure S1. Evidence was prioritized in the order: guidelines and formal consensus; systematic reviews and meta-analyses with certainty assessment; individual RCTs, giving equal weight to neutral or negative trials; controlled observational studies; mechanistic studies. For each major indication we summarize the direction and magnitude of effect, the comparator and follow-up, the reported certainty and principal risk-of-bias concerns (Section 4), and—separately from the evidence—our own interpretation, which is explicitly flagged as hypothesis. A narrative design was chosen because the central questions concern mechanisms and sources of heterogeneity that pooled estimates alone do not capture; this choice does not permit formal quantitative synthesis, and the review should not be read as one.

## 3. Matching Biology: Composition, Classification, and the Standardization Imperative

### 3.1. The Biological Cargo of Platelet Concentrates

Platelets are not passive carriers of growth factors; they are active immunomodulatory and reparative cells. Their alpha granules release platelet-derived growth factor (PDGF), transforming growth factor-β (TGF-β), vascular endothelial growth factor (VEGF), insulin-like growth factor-1 (IGF-1), epidermal growth factor, and fibroblast growth factor, which together drive angiogenesis, chemotaxis, matrix synthesis, and cell proliferation [9,10]. Dense granules and the platelet releasate additionally deliver bioactive lipids, serotonin, adenosine nucleotides, and a spectrum of chemokines that shape local inflammatory tone. Critically, the composition of the delivered product is not fixed by the label “PRP”: it depends on the donor, the harvest, the preparation protocol, and the biology of the recipient tissue [9,41]. Two cell types dominate the biological debate. Leukocytes—particularly neutrophils—contribute both pro-inflammatory and matrix-degrading activity but also participate in the neutrophil–macrophage crosstalk that governs the transition from inflammation to repair [17]. Red blood cells are generally discouraged in intra-articular preparations because of their association with joint inflammation [41]. These considerations underlie the distinction between leukocyte-rich (LR-PRP) and leukocyte-poor (LP-PRP) formulations. LP-PRP is commonly preferred for intra-articular use and LR-PRP is argued by some to suit recalcitrant tendinopathy, but this joint-versus-tendon heuristic rests on limited comparative data, and leukocyte and platelet concentrations alone do not reliably explain clinical outcomes [17]. The principal growth factors and their actions are summarized in Table 3 and, in simplified form, in Figure 1; the biological effect of each mediator is dose-, time-, tissue-, and microenvironment-dependent, and the linear cascade shown is a didactic simplification of a highly interactive network.

### 3.2. Product Variability Is the Field’s Central Confounder

The magnitude of preparation-driven variability is easily underestimated. In a characterization study of 335 knee-OA patients, the mean platelet concentration achieved in PRP was 1.26 × 10^6^/µL—roughly 6.3-fold over whole blood—but fold-enrichment varied inversely with baseline platelet count, ranging from 4.2-fold in patients with high baseline counts to 6.9-fold in those with low counts [42]. The same study documented that leukocyte subpopulations shift from a neutrophil-predominant profile in whole blood to a lymphocyte-predominant profile in PRP [42]. In other words, two patients treated with the identical device and protocol receive biologically different products, and a single reported “dose” is demonstrably inconsistent. Reviews of PRP mechanism and preparation reach the same conclusion: the absence of a standardized protocol produces a spectrum of final products whose comparability across trials is questionable [6,9,10]. This variability is a confounder of the evidence, but it is not the only one; trial-level sources of heterogeneity (Section 1) act in addition to it.

### 3.3. From Ad Hoc Labels to a Rational Classification—And the ISTH Transition

A succession of classification systems has attempted to impose order: PAW (platelets, activation, white cells), the Dohan Ehrenfest classification separating pure PRP, leukocyte-rich PRP, pure platelet-rich fibrin and leukocyte-and-platelet-rich fibrin (L-PRF), and later DEPA and MARSPILL schemes that added dose, efficiency, purity, and activation descriptors [24,43]. Each captured part of the biology; none achieved universal adoption, and their proliferation itself became an obstacle to cross-study synthesis [43]. A 2024 review tabulated eight such schemes (PAW, the Sports Medicine classification, PLRA, DEPA, MARSPILL, the ISTH system, and the six-digit coding system of Kon et al.), none of which has been adopted universally [9]. Among them, the Scientific and Standardization Committee of the International Society on Thrombosis and Haemostasis (ISTH), through its Platelet Physiology Subcommittee, convened a working party to produce consensus guidance for the use of platelets in regenerative medicine and proposed a classification that also accommodates newer activation techniques, including freeze–thaw preparations [23]. Notably, that working party stated that the available data were insufficient to develop evidence-based guidelines and therefore used the RAND appropriateness method to reach formal consensus on 45 statements—a fact that underscores rather than resolves the standardization gap. Situating a biology-matched framework within a shared, reproducible vocabulary of this kind—whatever scheme ultimately prevails—is nonetheless the precondition for any precision strategy, because a therapy that cannot be specified cannot be matched, and because unreported composition is itself a major source of the heterogeneity discussed throughout this review.

### 3.4. Beyond First-Generation PRP: A Widening—And Heterogeneous—Product Landscape

The biology axis also spans generations and sources of product, and these must not be conflated. Second-generation autologous platelet concentrates such as L-PRF are produced by a single centrifugation without anticoagulant or exogenous activator, yielding a three-dimensional fibrin scaffold that releases growth factors more gradually than liquid PRP but demands immediate handling because clotting begins in the collection tube [44]. Autologous protein solution, a PRP derivative enriched in anti-inflammatory cytokines and growth factors, and autologous conditioned serum extend the blood-derived family further [45]. Platelet- and marrow-derived biologics additionally exhibit antimicrobial activity through their leukocyte and antimicrobial-peptide content, a property of interest in pain medicine that likewise depends on unstandardized composition [46].

Terminology for marrow- and cell-based products requires particular care. BMAC is a heterogeneous, minimally processed concentrate of nucleated cells, platelets, and plasma in which connective-tissue progenitors are a small and highly variable minority. The classic non-union series quantified this directly: iliac-crest aspirates contained on average 612 ± 134 colony-forming progenitors per cm^3^ before concentration and 2579 ± 1121 per cm^3^ after it, with a range from 60 to 6120 per cm^3^; union was obtained in 53 of 60 patients, all of whom received grafts containing more than 1500 progenitors per cm^3^, whereas the seven failures received a mean of 634 per cm^3^ [25]. BMAC is therefore not equivalent to a defined, culture-expanded mesenchymal stromal cell (MSC) product, and clinical benefit from a marrow concentrate cannot be attributed to an MSC-mediated regenerative effect without direct evidence. Throughout this review we use “mesenchymal stromal cell” (the term recommended by the International Society for Cell & Gene Therapy) rather than “stem cell” unless quoting a source, and we treat BMAC, expanded MSCs, adipose-derived fractions, and perinatal products as distinct classes with distinct biology, evidence, and regulatory status (Section 9). Allogeneic and perinatal sources have entered practice: umbilical cord–derived PRP is reported to offer a more consistent, high–growth-factor composition than autologous preparations [47], and platelet- and MSC-derived extracellular vesicles are proposed as cell-free payloads (Section 7). Each product occupies a different position on the spectrum from pro-inflammatory to immunomodulatory and from scaffold-forming to freely diffusible—differences that would only become clinically actionable once shown, in comparative trials, to matter for the pathology in question. The preparation steps that generate these products, and the compositional layers each one draws upon, are shown in Figure 2.

## 4. Matching Pathology: Evidence Across Musculoskeletal Compartments

If the biology axis asks what is injected, the pathology axis asks whether that biology fits the mode of failure of the target tissue. Degenerative cartilage, tendinopathic collagen, an inflamed fascial enthesis, a degenerating disc, and a stalled fracture callus present distinct microenvironments, and the evidence base for each differs markedly in size, quality, and direction. In this section we summarize, for each indication, the best available comparative evidence together with its comparator, follow-up, effect magnitude together with what can and cannot be inferred from it, reported certainty, and principal risk-of-bias concerns; neutral and negative trials are given the same weight as positive syntheses. Table 4 consolidates this appraisal and assigns each indication–product pair a readiness category, and Figure 3 visualizes the evidence landscape. The “implications” offered are our interpretation and are flagged as hypotheses, not recommendations.

**Figure 3:**
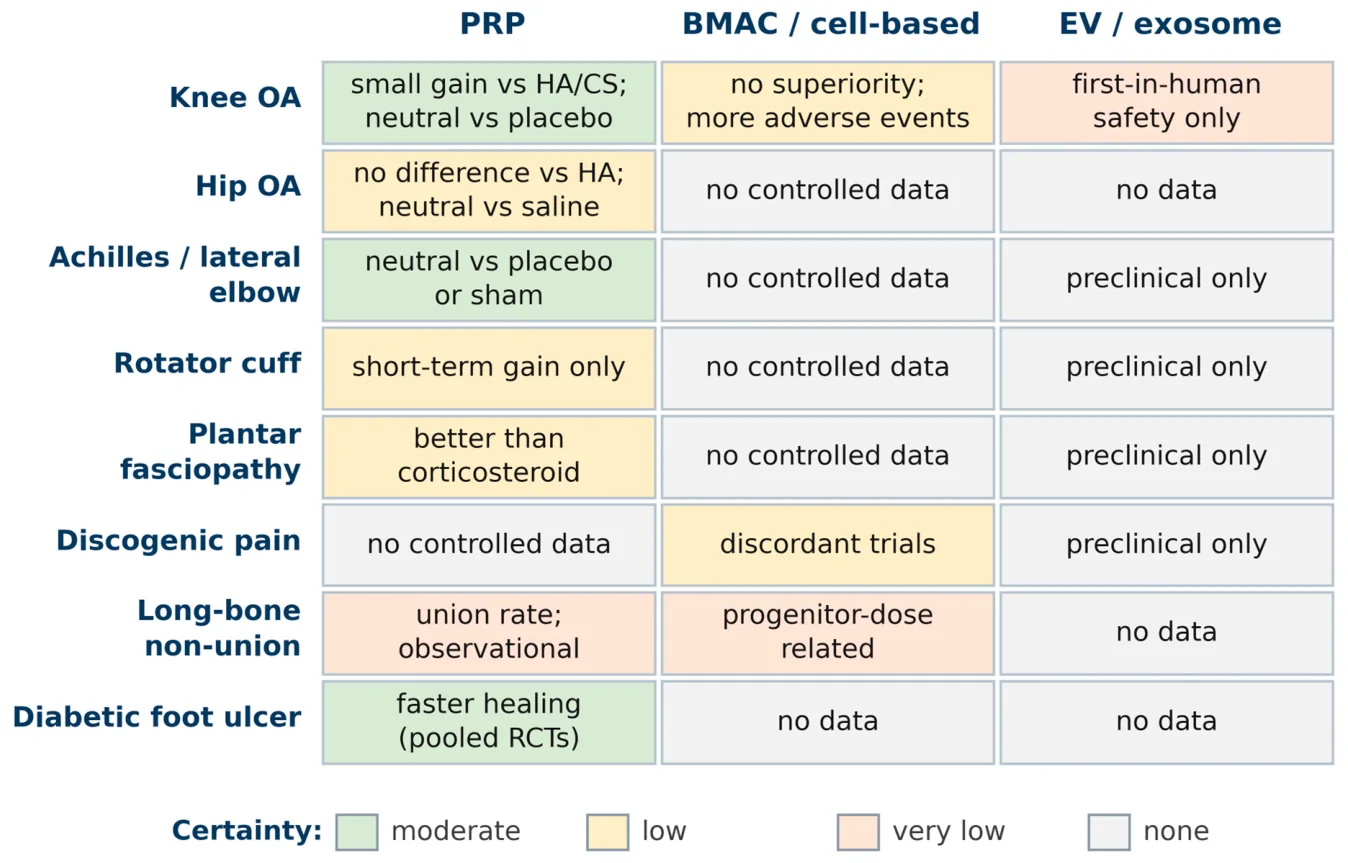
Evidence landscape for orthobiologics by indication and product class. Cells are shaded by the approximate certainty of the best available comparative evidence and annotated with the direction of the primary finding against the most rigorous comparator; “neutral” denotes an adequately controlled trial showing no difference from placebo or sham. The underlying sources are, by row: knee OA—meta-analysis of 42 randomized trials against hyaluronic acid or corticosteroid and the placebo-controlled RESTORE trial, the ESSKA-ORBIT Part 2 consensus and the pooled adverse-event analysis for cell-based products, and the first-in-human report for extracellular vesicles [5,7,11,28,29]; hip OA—meta-analysis of six trials against hyaluronic acid and one placebo-controlled trial of 60 patients [48,49,50]; Achilles and lateral-elbow tendinopathy—three placebo- or sham-controlled randomized trials [51,52,53]; rotator-cuff tendinopathy and plantar fasciopathy—pooled analyses against corticosteroid [18,19,54,55]; discogenic pain—the sham-controlled DREAM trial and the allogeneic mesenchymal precursor cell trial [20,56]; long-bone non-union—observational series in which union was related to the number of progenitors delivered [21,25]; diabetic foot ulcer—meta-analysis of 15 randomized trials [22]. The figure summarizes Table 4 and is descriptive, not a formal GRADE assessment.

**Table 4:** Critical appraisal of representative evidence for orthobiologics by musculoskeletal indication, with readiness classification.

Indication/Product	Representative Evidence (Design; n; Comparator; Follow-up)	Effect Magnitude and Its Interpretation	Certainty and Principal Risk-of-Bias Concerns	Maturity of the Evidence Base	Implications for Future Studies (Authors’ Interpretation)
Knee OA—PRP	MA of 42 RCTs, *n =* 3696, PRP vs. HA/CS, 1–12 mo [5]; narrative synthesis of 40 studies [6]; RESTORE RCT, *n =* 288, LP-PRP vs. saline, 12 mo [7]	WOMAC pain MD −0.74 vs. HA (scaling not stated in the source; no responder analysis; see Section 4.1 [31]); RESTORE: no difference vs. placebo in pain or cartilage volume	Moderate at best (GRADE); I^2^ > 90%; heterogeneous products; many trials unblinded or with unclear allocation; small-study effects; RESTORE low RoB	Many controlled trials; result small vs. active comparators and neutral vs. placebo	Hypothesis: effect may be larger in defined product/phenotype strata; requires biomarker-stratified, placebo-controlled trials. Guideline positions vary (Table 5)
Hip OA—PRP	MA of 6 level I–II RCTs, *n =* 408, PRP vs. HA, ~12 mo [48]; scoping review [49]; double-blind RCT, *n =* 60, PRP vs. saline, 6 mo [50]	No significant between-group difference vs. HA (WOMAC, VAS, HHS) and none vs. saline in the one placebo-controlled trial	Low; single small placebo-controlled trial; superiority designs not powered for equivalence; heterogeneous protocols; advanced structural stage	Few controlled trials; result neutral	Non-significance is not equivalence. Adequately powered, image-guided, placebo-controlled trials are the priority
Knee OA—BMAC/cell-based	ESSKA-ORBIT Part 2 consensus [28]; ESSKA safety MA (5853 cell-treated pts) [11]	Symptomatic benefit ≤ 12 mo in KL 1–3; no superiority over other injectables; no disease modification shown	Low; few RCTs; heterogeneous products (BMAC ≠ expanded MSC); highest AE proportion among injectables	Few controlled trials; no superiority shown	Second-line within trials/registries; separate BMAC from expanded MSC in future studies; report progenitor content
Tendinopathy—site-specific	MA 27 RCTs, *n =* 1779, PRP vs. CS, mixed sites [18]; rotator cuff MA 30 RCTs [54]; Achilles RCTs vs. saline/sham (*n =* 54; *n =* 240) [51,52]; lateral elbow RCT vs. saline [53]	PRP > CS at 3–6 mo (CS deteriorates late); PRP = placebo for Achilles (VISA-A) and lateral elbow (pain) at primary time points	Low–moderate; high heterogeneity when sites pooled; well-controlled neutral trials at two sites	Several controlled trials; neutral vs. placebo at the two best-studied sites	Analyse by tendon and time point; PRP may lack specific effect in some tendon phenotypes irrespective of stratification
Plantar fasciopathy	MA 24 RCTs, *n =* 1653, PRP vs. CS, 1–12 mo [19]; NMA 63 RCTs, *n =* 4170, multiple comparators [55]	PRP > CS at 3–6 mo (VAS) and 3–12 mo (AOFAS); NMA ranks PRP highest for long-term function; rankings low-certainty	Low–moderate; indirect comparisons; imprecision; few placebo-controlled trials	Several controlled trials; no placebo-controlled evidence	Tailoring by symptom duration/goal is plausible; needs placebo-controlled trials and standardized fascia-thickness outcomes
Discogenic/spinal	DREAM phase IIB RCT, *n =* 52, intradiscal BM-MSC vs. sham, 6 mo [20]; allogeneic MPC RCT, *n =* 100, 36 mo [56]	DREAM: structural change (disc-height index) without clinical advantage over sham. MPC trial: superiority over controls on pain, disability and composite endpoints through 36 months, in an analysis adjusted for post-treatment interventions; no difference in modified Pfirrmann score	Low; small samples; differing cell source, disease stage and follow-up; adjusted analysis population and industry sponsorship in the positive trial	Two controlled trials; results discordant	Discordance is itself informative: it may reflect cell source, disease stage or analysis choices—the kind of question a stratified trial is designed to answer
Bone—delayed/non-union	SR 20 studies, *n =* 749, mostly non-randomized [21]; progenitor-dose series [25]	Trend to higher union with biologic augmentation; union related to progenitor dose	Very low–low; observational; heterogeneous	Observational only	Characterize and report progenitor content; comparative RCTs needed
EV/exosome products (all sites)	Preclinical SR (28 rat studies) [57]; single first-in-human ascending-dose RCT [29]	Consistent histological benefit in rodents; early human safety with limited efficacy signal	Very low for efficacy; no comparative human data	Predominantly preclinical	Not a current alternative to PRP; requires MISEV-compliant characterization, GMP manufacture, dose-finding and controlled trials (Section 7)

MA, meta-analysis; NMA, network meta-analysis; SR, systematic review; RCT, randomized controlled trial; CS, corticosteroid; HA, hyaluronic acid; MD, mean difference; MCID, minimal clinically important difference; KL, Kellgren–Lawrence; BM-MSC, bone marrow mesenchymal stromal cell; MPC, mesenchymal precursor cell; EV, extracellular vesicle; AE, adverse event; RoB, risk of bias. Three properties are reported separately and should not be conflated: the certainty of the evidence, the direction of the result against the most rigorous comparator, and the maturity of the evidence base. Maturity categories describe how much controlled evidence exists—many controlled trials; few controlled trials; single or small controlled trials; predominantly preclinical—and say nothing about whether the intervention works; a mature evidence base with a neutral result is not a treatment supported by evidence. Clinical applicability is given separately in Section 8.2. The final column is the authors’ interpretation and does not constitute a clinical recommendation.

### 4.1. Osteoarthritis: The Knee, and the Thinner Evidence Beyond It

Knee OA generates the largest body of orthobiologic evidence and, correspondingly, the sharpest controversy. An updated meta-analysis of 42 RCTs (3696 patients) found that intra-articular PRP produced statistically greater pain relief than HA—WOMAC pain mean difference −0.74 (95% CI −1.02 to −0.46; I^2^ = 94%)—and outperformed corticosteroid, with the largest advantage at approximately six months [5]. Whether such a pooled difference is clinically relevant cannot be settled as simply as is often assumed. The source synthesis does not state the scaling of the WOMAC pain subscale from which the pooled estimate was derived, and the most widely cited threshold in this field—the minimal clinically important improvement (MCII) of Tubach et al.—was derived for pain measured on a visual analogue scale (−19.9 mm in knee OA) and for function on the WOMAC function subscale (−9.1), not for the WOMAC pain subscale [31]. Two further distinctions matter. The MCII is an individual-level threshold, intended to convert trial results into the proportion of patients who improve; it is not a benchmark against which a between-group mean difference should be read, because a mean difference below an individual threshold is compatible both with a uniformly negligible effect and with a minority of substantial responders. A difference against an active comparator is not a difference against no treatment. What can be said is that the pooled advantage is small in absolute terms, rests on estimates with I^2^ above 90%, and is not accompanied by responder analyses; whether it corresponds to a benefit patients would notice remains undetermined by the available syntheses. A comprehensive 2025 narrative synthesis of 40 higher-quality studies reached a compatible, qualified conclusion: PRP—particularly LP-PRP—offers greater pain relief and function than HA and corticosteroid in mild-to-moderate disease (Kellgren–Lawrence I–III), but heterogeneity in formulation, regimen, and follow-up limits comparability, several placebo-controlled trials fail to show superiority over saline beyond 6–12 months, and the overall GRADE certainty is moderate at best [6].

The most rigorous placebo-controlled evidence is neutral. The RESTORE trial randomized 288 participants with symptomatic mild-to-moderate medial knee OA to three weekly injections of a commercial leukocyte-poor PRP or saline, with participant, injector, and assessor blinding and 93% 12-month follow-up; PRP was no better than placebo for 12-month knee pain (difference −0.4 points on an 11-point scale; 95% CI −0.9 to 0.2) or medial tibial cartilage volume (difference −0.2%; 95% CI −1.9% to 1.5%), and 29 of 31 secondary outcomes showed no between-group difference [7]. In a surgical context, a small single-blind randomized study (*n* = 29) of PRP augmentation of arthroscopic microfracture in patients over 50 found improvement in both arms but no significant between-group difference in WOMAC (*p* = 0.70) or Lysholm (*p* = 0.94) at 24 weeks [8]. Two interpretations of the discordance between active-comparator meta-analyses and placebo-controlled trials are possible and are not mutually exclusive. One is that PRP has a modest, real effect that is comparable to or slightly greater than that of HA and corticosteroid but that is small relative to the contextual response to intra-articular injection itself. The other—the precision hypothesis—is that a well-characterized product in an appropriately selected population would show a larger effect than the average across heterogeneous products and populations. RESTORE, which used a single standardized LP-PRP and a reasonably homogeneous KL 2–3 population, offers little direct support for the second interpretation and should not be reframed as evidence that a better phenotype simply needs to be found. We regard the precision hypothesis for knee OA as plausible but untested, and we note that it is fully compatible with PRP ultimately proving to have limited clinical utility in some or all subgroups.

Viewed as a whole, the knee-OA research ecosystem illustrates the central tension. Across RCTs, PRP generally shows superior short- to intermediate-term outcomes to corticosteroid and HA in early-to-moderate disease, with benefits sometimes persisting to one year; but fragility-index analyses indicate that many positive trials are only modestly robust, systematic reviews have at times overstated the magnitude or certainty of benefit, and variation in platelet and leukocyte concentration alone does not account for differences in outcome [17]. Patient-level factors—metabolic health, systemic inflammatory status, structural disease phenotype, and, as discussed in Section 5.4, pain-processing phenotype and psychological status—are candidate explanatory variables [17]; whether they are true effect modifiers remains to be shown. A roadmap linking product characterization, standardized reporting, patient stratification, and objective outcome reporting has been proposed as the route from “a generic injection toward a tailored regenerative therapy” [17]; we endorse it as a research agenda rather than as current practice.

The evidence outside the knee is markedly thinner, and this asymmetry deserves to be stated rather than smoothed over. For hip OA, a meta-analysis of six level I–II randomized trials (211 patients receiving PRP, 197 receiving HA) did not demonstrate a significant between-group difference in WOMAC, VAS, or Harris Hip Score improvement, including in a subanalysis restricted to leukocyte-poor preparations [48]; a subsequent scoping review reached the same conclusion, contrasting the relatively consistent knee literature with inconsistent hip results [49]. Absence of a significant difference is not equivalence: these trials were designed and powered for superiority, and none was an equivalence or non-inferiority study. Placebo-controlled evidence at this site is limited to one small double-blind trial in which 60 patients with Kellgren–Lawrence grade 2–3 hip OA received three weekly ultrasound-guided injections of PRP or saline; over six months PRP showed no significant advantage over saline on the visual analogue scale, WOMAC total score, or most SF-36 domains [50]. That single trial is too small to exclude a modest effect, but it is the only placebo-controlled evidence available and it is neutral. Two features of the hip may be relevant to the pathology axis: the joint is deep and its injection accuracy without image guidance is poor (Section 8.3), and hip OA is typically diagnosed at a more advanced structural stage than knee OA. For glenohumeral, first carpometacarpal, ankle, and facet-joint OA the controlled evidence is smaller still and is insufficient to support indication-specific conclusions. We therefore treat knee OA as the reference indication throughout this review and flag extrapolation to other joints as unsupported—a limitation of the field rather than a matter of interpretation.

### 4.2. BMAC and Cell-Based Therapy in Osteoarthritis

Cell- and tissue-based therapies sit at an earlier stage of evidence than blood-derived products: their development pipelines are longer and costlier, and although several products have demonstrated clinical safety, the number of RCTs and meta-analyses remains considerably smaller and concentrated in knee OA [3]. Two categories must be distinguished (Section 3.4). Point-of-care BMAC and microfragmented or enzymatically processed adipose tissue are heterogeneous, minimally processed concentrates; expanded MSC products are defined, culture-manufactured biologics. For BMAC, findings remain inconsistent and long-term efficacy relative to PRP or autologous conditioned serum is not established, compounded by variability in harvest site, aspiration technique, and concentration protocol that affects progenitor yield [25]. The ESSKA-ORBIT Part 2 consensus on cell-based therapy for knee OA, developed by a multidisciplinary European panel with statements graded by level of evidence and expert agreement, concluded that cell-based therapy provides benefit in pain and function for up to 12 months in Kellgren–Lawrence grade 1–3 disease, with lesser benefit in selected grade 4, but that because of limited high-quality studies and the absence of clear superiority over other injectables it should be regarded as a second-line option [28]. The ESSKA safety meta-analysis further found that cell-based therapies had the highest rate of patients with at least one adverse event among the intra-articular injectables examined (14.7%), although with the caveats discussed in Section 4.8 [11]. We interpret this evidence as supporting cautious, second-line use within trials or registries rather than routine practice; the frequently invoked mechanistic rationale (MSC-mediated regeneration) is not demonstrated in humans by any of the cited studies.

### 4.3. Tendinopathy—Site-Specific Biology and a Mixed Trial Record

“Tendinopathy” is not a single condition. Insertional and midportion Achilles tendinopathy, lateral epicondylalgia, patellar tendinopathy, gluteal tendinopathy, and rotator-cuff disease differ in vascularity, loading environment, the balance of degenerative and inflammatory features, the relevant comparators, rehabilitation pathways, and injection risks, and the evidence for orthobiologics differs correspondingly. Pooled analyses that combine them therefore risk the very error this review argues against. A 2025 systematic review and meta-analysis of 27 RCTs (1779 patients) comparing PRP with corticosteroid across mixed tendinopathies found that pooled effects were site- and time-dependent: corticosteroid tended to be superior at one month, whereas PRP was associated with better VAS and DASH scores at three and twelve months in lateral epicondylalgia, with better VAS and AOFAS scores at six months (but not at one or three months) in plantar fasciopathy, and with lower VAS in rotator-cuff disease at three months, with long-term efficacy unconfirmed and substantial heterogeneity and risk of bias in the included trials [18]. When the evaluation is limited to the rotator cuff, a synthesis of 30 RCTs (~2500 patients) found that PRP reduced pain and improved function in the short term relative to placebo and corticosteroid, but that benefits beyond twelve months were limited and retear rates after repair were similar to controls [54].

The placebo-controlled trial record is sobering and must be given equal weight. In chronic midportion Achilles tendinopathy, a double-blind RCT of a single PRP injection versus saline, both with eccentric exercise, found no difference in VISA-A score at 24 weeks [51], and the larger ATM trial (*n =* 240) found no difference between PRP and sham injection in VISA-A at six months [52]. In lateral epicondylalgia, a three-arm double-blind RCT found that neither PRP nor glucocorticoid was superior to saline for pain at three months, although glucocorticoid reduced tendon thickness and Doppler activity [53]. These well-conducted neutral trials indicate that, for at least two common tendinopathies, PRP does not outperform placebo on the primary outcome at the time points tested. The positive medium-term signals in comparative meta-analyses may reflect corticosteroid’s known late deterioration rather than an intrinsic PRP benefit. The lesson is twofold: site-specific analysis is required, and the precision hypothesis must accommodate the real possibility that PRP has little or no specific effect in some tendon phenotypes irrespective of stratification.

### 4.4. Plantar Fasciopathy—Matching Intervention to Symptom Horizon

Plantar fasciopathy—the term we use throughout in preference to “fasciitis”, because the chronic condition is predominantly degenerative rather than inflammatory—is among the most common causes of heel pain and is addressed by a large literature of overlapping conservative and minimally invasive options. Its epidemiology is, however, less firmly established than is usually implied: a systematic review of 96 systematic reviews found that most had substantial heterogeneity, that evidence on epidemiology specifically was lacking, and that among risk factors only a raised body-mass index was consistently supported [59,60]. In a meta-analysis of 24 RCTs (1653 participants), PRP was associated with better VAS pain than corticosteroid injection at three and six months and better AOFAS function at three, six, and twelve months, with no difference at one month [19]; a retrospective series of 140 conservative-therapy failures likewise reported improvement at three months after PRP, without a control group [61]. A 2026 network meta-analysis of 63 RCTs (4170 participants) ranked interventions across horizons: botulinum toxin A ranked highest for short-term pain and fascia thickness, corticosteroid for short-term function, prolotherapy for sustained pain relief, PRP for long-term function and fascia thickness, and extracorporeal shockwave therapy performed consistently across domains [55]. Network rankings of this kind rest on indirect comparisons, imprecise estimates, and low-certainty evidence for several nodes, and a “best” ranking at a time point should not be read as a demonstrated superiority; nevertheless, the authors’ conclusion that no single agent dominates and that treatment might reasonably be tailored to symptom duration and therapeutic goal [55] is consistent with—though not proof of—the pathology-matching principle. Corticosteroid’s early advantage and later attrition, and PRP’s slower but more durable trajectory in these analyses, are the main practical signal.

### 4.5. Discogenic and Spinal Pain—A Young Field with Discordant Controlled Trials

Chronic low back pain of discogenic origin is a domain in which matched orthobiologics could, in principle, add value because the alternatives are either non-specific or invasive; it is also a domain in which the two available controlled trials point in opposite directions. The phase IIB, double-blind DREAM study randomized 52 patients with chronic low back pain due to moderate-to-advanced multilevel disc degeneration to autologous intradiscal bone marrow–derived MSCs or a sham procedure [20]. The cell injection was well tolerated and was accompanied by structural change—a significant increase in disc-height index and a non-significant trend toward improved T2 relaxation times, with modified Pfirrmann grade improving transiently at three months but returning to baseline at six—yet pain, disability, and quality-of-life scores improved similarly in both arms, with no significant clinical advantage over sham at six months and slightly greater improvement in ODI and SF-36 physical component in the sham group [20]. A larger and longer randomized controlled study reached a different conclusion. One hundred patients with chronic low back pain and moderate degeneration were randomized to two doses of allogeneic mesenchymal precursor cells with hyaluronic acid, to hyaluronic-acid vehicle, or to saline; over 36 months the cell-treated groups were superior to controls on pain and disability and on responder and composite endpoints at various time points [56]. Three qualifications belong with that result: the efficacy analysis was adjusted for the confounding effect of post-treatment interventions rather than analysed as randomized throughout, the modified Pfirrmann score did not differ between groups, and the trial was sponsored by the manufacturer. The two trials differ in cell source (autologous marrow versus allogeneic precursor cells), in disease stage (moderate-to-advanced multilevel versus moderate single-level), in follow-up (6 versus 36 months) and in analysis population, so they are not straightforwardly contradictory; but neither can be read as confirming the other. Three interpretations of DREAM are equally admissible: that the structural signal was real but not clinically meaningful; that the intervention is ineffective for this population; or that a better-selected population (earlier degeneration, biochemically confirmed discogenic pain) might respond. The last is the precision hypothesis; it is a rationale for a stratified trial, not a demonstrated explanation, and it should not be allowed to soften the primary finding that a well-controlled trial was neutral. We also note that structural regeneration, even when demonstrable, cannot substitute for clinically meaningful improvement in pain, function, or the need for subsequent intervention when the therapeutic goal is chronic pain relief.

### 4.6. Bone: Delayed Union and Non-Union of Long-Bone Fractures

Impaired fracture healing is a pathology of a missing favorable biological microenvironment, and orthobiologics are conceptually suited to restore it; the evidence base is, however, small and largely observational. A systematic review of orthobiologic strategies in delayed union and non-union of adult long bones (20 studies; 749 patients) described the combined use of osteoinductive growth factors, osteogenic cells, and osteoconductive scaffolds and reported that biologic augmentation—most often PRP—tended toward better union rates than procedures without biologic factors, but on the basis of heterogeneous, mostly non-randomized studies with variable follow-up [21]. The classic percutaneous marrow-grafting series showed that union was obtained only in patients whose grafts exceeded roughly 1500 colony-forming progenitors per cm^3^, a concentration that unconcentrated iliac-crest marrow rarely reached [25]—an early and specific example of the product-characterization principle. Here the matching concept is almost literal: the product should furnish whichever element of the “diamond concept”—signal, cells, or scaffold—the failing osseous environment lacks. The evidence remains insufficient for comparative recommendations.

### 4.7. Extrapolating from Wound Healing

Diabetic wound healing, though outside the joint, offers unusually clean evidence about host biology as a determinant of orthobiologic outcome because the endpoint (ulcer closure) is unambiguous. A meta-analysis of 15 RCTs (1010 patients) found that PRP improved complete healing (RR 1.53) and shortened healing time compared with conventional care [22]. The more instructive signal is who fails: in a prospective cohort of 182 patients, multivariate analysis identified body-mass index ≥ 30 kg/m^2^ and diabetes duration ≥ 20 years as independent predictors of PRP failure, with smoking and impaired renal function also associated [62]. These are prognostic associations in a treated cohort, not treatment-effect modifiers (Section 5.1), and their transfer to intra-articular and spinal indications is plausible but unproven.

### 4.8. Safety Across Indications—And the Limits of Pooled Comparisons

Any decision to offer an orthobiologic weighs an uncertain benefit against a quantifiable risk, so the comparative safety data deserve the same scrutiny as the efficacy data—and the same caution about what pooling can and cannot support. The ESSKA Orthobiologics Initiative safety meta-analysis pooled 559 studies reporting adverse events in 76,061 patients treated with intra-articular corticosteroid, HA, PRP, or cell-based therapies for knee OA [11]. The proportion of patients with at least one adverse event was 8.7% for PRP, 10.5% for HA, 11.0% for corticosteroid, and 14.7% for cell-based therapies, with severe adverse events in 0.3% of PRP-treated patients and infection in 0.3%; most PRP-related events were transient local reactions [11]. These figures are reassuring for autologous PRP, but they should not be read as a definitive comparative ranking. Adverse-event definitions, ascertainment methods, follow-up duration, study design, reporting completeness, and patient selection differed across the pooled studies, and proportions aggregated across hundreds of heterogeneous studies are susceptible to reporting bias in ways that a randomized head-to-head comparison would not be. Two cautious implications follow. First, the safety of point-of-care autologous PRP cannot be extrapolated to culture-expanded cells, perinatal products, or extracellular vesicle preparations, each of which carries distinct theoretical risks (immunogenicity, thrombogenicity, tumorigenicity, transmission) and much smaller safety datasets. Second, the higher adverse-event proportion observed for cell-based therapies belongs on the risk side of any decision; it does not, on its own, establish a treatment sequence, since these are not head-to-head comparisons and the products differ in the populations and settings in which they have been studied. Across the non-articular indications reviewed here—tendon, fascia, bone, and disc—serious orthobiologic-related adverse events were uncommon in the cited trials, but safety reporting was inconsistent and warrants standardization alongside composition reporting.

## 5. Matching Patient Phenotype: Prognostic Factors, Candidate Predictors, and Response Modifiers

The third axis asks which patient, within a given indication and receiving a given product, is likely to benefit. The evidence here is the newest and least mature, and it requires a terminological discipline that the orthobiologic literature has often lacked. A prognostic factor is associated with outcome irrespective of treatment; a predictive biomarker (treatment-effect modifier) identifies patients who benefit differentially from one treatment relative to another and can only be established by demonstrating a treatment-by-biomarker interaction in a comparative study [30]. Almost all the variables discussed below were identified in single-arm or single-treatment cohorts and are therefore, at present, prognostic factors or response correlates; none has been shown to modify the effect of an orthobiologic relative to an appropriate comparator such as HA, corticosteroid, exercise, or placebo. A baseline KOOS, PROMIS score, or MRI pattern that predicts outcome after PRP may equally predict outcome after any intervention and therefore does not, by itself, tell a clinician whether to choose PRP. We use “prognostic factor,” “response correlate,” “candidate predictive biomarker,” and “validated effect modifier” accordingly, and we summarize the readiness of each phenotyping tool in Section 8.2.

### 5.1. Structural Phenotype and Imaging

Baseline joint structure is a prognostic factor. In a retrospective analysis of 75 knee-OA patients receiving a single injection of extensively characterized pure PRP, 63% were responders (≥10-point KOOS improvement) at six months; non-response was associated with three-compartment involvement on baseline MRI and, counterintuitively, with a higher delivered platelet dose [63]. The study lacked a comparator, so it identifies patients with a poorer prognosis after PRP rather than patients who would do better with an alternative; it does, however, caution against the assumption that more product is better in advanced multi-compartment disease. Advanced quantitative imaging—T2 mapping, diffusion tensor imaging, dynamic contrast-enhanced MRI, and molecular PET—has been proposed to phenotype tissue health beyond the Kellgren–Lawrence grade [2]; these remain research tools, and structural severity correlates only modestly with symptoms in both knee OA and chronic low back pain, which limits the value of structure-only stratification for a pain outcome.

### 5.2. Inflammatory Phenotype

A patient’s inflammatory state is a candidate predictive axis but the evidence is preliminary. Elevated baseline synovial-fluid interleukin-6 has been associated with poorer response to PRP in knee OA in small cohorts, suggesting inflammatory and non-inflammatory endotypes that might warrant different products or sequencing [2]. This observation deserves careful interpretation: if synovial inflammation marks a poorer response to PRP, then PRP should not be preferentially directed toward “inflammatory” joints, and no such pairing is proposed in Table 6. Peripheral-blood inflammatory indices derived from a routine complete blood count—neutrophil-to-lymphocyte ratio, platelet-to-lymphocyte ratio, systemic immune-inflammation index, and monocyte-to-lymphocyte ratio—are under prospective evaluation as inexpensive correlates of PRP response, building on signals that peripheral inflammatory phenotype tracks with outcome [2,17]. That platelet and leukocyte dose appear not to explain outcomes [17] while patient inflammatory status may, is a reason to study the patient axis; it is not yet evidence that inflammatory status should direct product choice.

### 5.3. Host and Metabolic Factors

As the diabetic-wound data show, host biology sets a ceiling on what an autologous product can achieve [62]. Obesity, long-standing diabetes, smoking, renal impairment, and the broader metabolic syndrome plausibly degrade both the quality of the harvested product and the responsiveness of the target tissue, and metabolic OA is a recognized phenotype that may behave differently from post-traumatic or primarily mechanical disease [4,17]. Documenting metabolic comorbidity before injection is inexpensive and implementable now; whether it modifies the relative benefit of PRP over alternatives is unknown.

### 5.4. Pain-Processing and Psychosocial Phenotype

A precision framework for chronic pain that considers MRI, cytokines, blood counts, and metabolic status but not pain mechanisms is incomplete, and this omission is common in the orthobiologic literature. As noted in Section 5.1, structural severity correlates only weakly with symptom intensity, and a substantial proportion of patients exhibit features of nociplastic pain—altered central processing with widespread hyperalgesia, temporal summation, and impaired conditioned pain modulation—that predict pain severity independently of joint pathology [64,66]. Central sensitization, neuropathic-like symptoms, pain catastrophizing, depression, anxiety, sleep disturbance, treatment expectation, prior pain duration, and socioeconomic context are established prognostic factors for outcome after MSK interventions of all kinds, and the machine-learning study discussed in Section 5.6 found that, among the ten pre-injection variables evaluated—all of them demographic or patient-reported—baseline PROMIS mental and physical health scores were the most influential predictors of achieving the MCID after PRP, and that removing them reduced the model to chance [65]. That study did not include structural or product-composition variables at all, which is itself informative about how the field has framed the prediction problem. These findings argue for integrating validated pain-phenotype instruments (for example, the Central Sensitization Inventory, painDETECT, the Pain Catastrophizing Scale, PROMIS mental health) into the phenotype axis. Their implication for orthobiologic selection is, again, prognostic: a patient with a predominantly nociplastic phenotype is likely to respond less to any peripherally directed injection, which argues for adequate assessment and multimodal care rather than for a particular biologic. We regard pain-phenotype assessment as implementable now and as a required stratification variable in future trials (Section 11).

### 5.5. Intraprocedural Signals: Pathological-Site Pain—A Hypothesis-Generating Observation

Some potential signals emerge at the point of care. In a single-center study of 32 patients with early knee OA treated with autologous protein solution combined with HA, those who experienced pain at the pathological site during injection had better one-year outcomes across pain, stiffness, function, and satisfaction, with an overall OMERACT–OARSI responder rate of 65.6% [45]. The observation is intriguing, but its interpretation is speculative. Pain during injection could reflect accurate targeting of a symptomatic lesion, but it could equally reflect intra-articular pressure and volume, synovitis, generalized nociceptive sensitivity (Section 5.4), anxiety, needle position, or technique; the cohort was small, uncontrolled, and treated with a combination product rather than a broad orthobiologic population; and no replication exists. We therefore present pathological-site pain as a hypothesis-generating procedural signal that merits prospective recording and adjusted analysis, not as a validated predictor that should influence treatment selection. It is included in Table 7 in the “experimental” tier.

### 5.6. Machine Learning as an Integrator

Because candidate factors are numerous, interacting, and partly non-linear, machine learning is a natural integrator. An explainable model developed on 191 knee-OA patients predicted achievement of the MCID six months after PRP with an area under the ROC curve of 0.81 (95% CI 0.65–0.94); an Explainable Boosting Machine was chosen for interpretability, baseline PROMIS mental and physical scores together with the KOOS-JR score were the most influential predictors, and excluding the PROMIS scores collapsed performance to near-chance (AUC 0.51) [65]. The authors were appropriately cautious: the model was developed in a single center, was not externally validated, had wide confidence intervals, and—because all patients received PRP—predicts prognosis after PRP rather than benefit from PRP relative to alternatives [65]. In adjacent interventional practice, a general-purpose large language model applied zero-shot to spinal cord stimulation candidacy agreed only fairly with the reference multidisciplinary decision (weighted κ = 0.29) [69]—a reminder that a model not calibrated for the specific decision will not reproduce expert selection. Such tools may in future complement clinical judgment, consistent with the broader application of artificial intelligence to phenotyping and outcome prediction across regenerative and neuromodulatory pain interventions [2,15], but they are research-stage today and will require external validation, calibration, and, ideally, testing within comparative trials in which the model’s recommendation is itself randomized.

## 6. The Guideline Landscape: Divergence and Its Several Explanations

Professional-society positions on injectable orthobiologics for knee OA differ, and the differences deserve a neutral reading. Divergence is sometimes read as evidence that undifferentiated use has “reached its ceiling.” That interpretation is one of several. Guidelines differ in their literature cut-off dates, evidence thresholds, grading systems (GRADE versus society-specific schemes), comparators of interest, definitions of clinically meaningful benefit, treatment of low-certainty evidence, and scope (knee only versus multiple joints); the same trials underlie a strong recommendation against, an “insufficient evidence” verdict, and a “limited” rating in favour, depending on the grading system, the threshold for acting under uncertainty, and the weight given to product heterogeneity as a reason to advise against rather than to qualify. Divergence is therefore consistent with product and population heterogeneity but is not proof of it, and it cannot be used as evidence for the precision hypothesis. Table 5 sets out the positions of five documents, verified against the primary sources with their publication years, wording, and strength or level of recommendation; the categories are not interchangeable and are reproduced as stated by each body.

Read side by side, the documents converge on two points and diverge on a third. They converge in finding the evidence for PRP to be heterogeneous and of limited certainty, and in not regarding any orthobiologic as disease-modifying. They diverge sharply on the practical inference, and the divergence is wider than a single reading of the evidence can explain—one body issues its strongest possible negative recommendation while another declines to recommend in either direction and a third treats PRP as a reasonable option: the ACR/Arthritis Foundation issued a strong recommendation against PRP for knee and/or hip OA—and, on the same reasoning, against stem-cell injections—citing “concern regarding the heterogeneity and lack of standardization in available preparations of platelet-rich plasma, as well as techniques used, making it difficult to identify exactly what is being injected” [32]; OARSI did not include PRP among its recommended treatments at any level for knee, hip, or polyarticular OA [33]; the AAOS assigned PRP a “limited” strength of recommendation, stating that it may reduce pain and improve function in symptomatic knee OA [34,58]; the 2026 VA/DoD guideline concluded that there is insufficient evidence to recommend for or against PRP for either knee or hip OA, assigning it the “neither for nor against” category [35]; and the ESSKA-ORBIT consensus went furthest in the opposite direction, regarding PRP as a valid and possibly first-line injectable option for Kellgren–Lawrence grade 1–3 knee OA—a position reached by formal consensus in which only 3 of 28 statements carried high-level scientific support and 19 rested on low-level evidence or expert opinion [27]. Broader instructional reviews reach a consistent meta-conclusion: meta-analyses report improvement in patient-reported outcomes with PRP in knee OA, lateral epicondylalgia, and plantar fasciopathy, but the included RCTs carry a high risk of bias driven by heterogeneous preparation protocols and inconsistent trial quality [3]. Non-standardized products applied to unstratified patients are one shared feature of that evidence; methodological weakness independent of product is another. Both are reasons for better trials.

## 7. Future Directions: Extracellular Vesicles and Standardized Allogeneic Products—Promise and Constraints

One response to the standardization problem is to move beyond whole-cell and whole-blood products toward defined, cell-free payloads. The rationale is coherent: a manufactured, characterized vesicle preparation could in principle be dosed and reproduced in ways an autologous concentrate cannot. The current evidence, however, is predominantly preclinical, and the terminology, manufacturing, and safety questions are substantial. We set out both sides.

### 7.1. Terminology and Characterization Standards

“Extracellular vesicle” (EV) is the umbrella term for membrane-bound particles released by cells; “exosome” denotes a specific endosomal-origin subpopulation whose biogenesis can rarely be demonstrated in a therapeutic preparation. The Minimal Information for Studies of Extracellular Vesicles (MISEV2023) guidelines recommend “EV” unless subcellular origin is proven and specify minimum requirements for source characterization, isolation and purification methods, quantification (particle number, protein, lipid), characterization of EV markers and non-EV contaminants, and functional assays [26]. Many orthobiologic EV studies, including those cited below, do not fully meet these requirements; procurement and characterization of platelet-derived EVs (PDEVs) in particular remain heterogeneous. We use “EV” throughout and “exosome” only when quoting a source.

### 7.2. Platelet-Derived Extracellular Vesicles

PDEVs carry a portion of the signaling cargo of the parent platelet in smaller, more stable, and potentially standardizable particles. Restorative effects have been reported in musculoskeletal and neural tissue in preclinical models. For the spine specifically, PDEVs ameliorated intervertebral disc degeneration in a rodent model by alleviating mitochondrial dysfunction in nucleus pulposus cells [67]—a mechanistically direct link to the clinical frontier of Section 4.5, but one that remains preclinical.

### 7.3. Mesenchymal Stromal Cell–Derived Extracellular Vesicles

MSC-derived EVs reproduce part of the anti-inflammatory, immunomodulatory, and pro-anabolic activity of their parent cells with greater stability and, in principle, lower immunogenicity [29,68]. A systematic review and meta-analysis of 28 studies in rat knee-OA models found consistent histological benefit across OARSI, Mankin, and ICRS scores, with subgroup signals favoring umbilical-cord and synovial-fluid MSC sources and biweekly dosing [57]; mechanistic work attributes these effects to macrophage repolarization, suppression of NF-κB and MAPK signaling, reduced IL-1β and TNF-α, protection of extracellular matrix, and up-regulation of chondrogenic markers with down-regulation of MMP-13 and ADAMTS5 [68]. Human data are limited to a single randomized, double-blind, ascending-dose study of umbilical-cord MSC–derived EVs for knee OA, preceded by chondrocyte toxicity testing, which reported no serious adverse events and “a certain degree” of improvement in clinical scores and MRI without a placebo comparison of efficacy [29]. This is a safety and feasibility signal, not evidence of comparative efficacy.

### 7.4. Constraints: Manufacturing, Dosing, Biodistribution, and Safety

The obstacles between this preclinical promise and a clinically usable product are considerable and should be stated plainly. Scalable manufacture under good manufacturing practice, batch-to-batch consistency, purity from co-isolated proteins and lipoproteins, potency assays that predict biological activity, storage stability, and particle quantification are unsolved for most candidate products [26]. Dose is undefined: rodent regimens do not translate directly, and the first-in-human study was an ascending-dose design without a validated dose–response relationship [29]. Biodistribution after intra-articular or intradiscal injection, retention time, and clearance are poorly characterized. Safety considerations specific to EVs include immunogenicity of allogeneic cargo, procoagulant activity (particularly for platelet-derived vesicles, which are physiologically prothrombotic), the theoretical transfer of oncogenic or pro-senescent cargo from parent cells, contamination and transmission risk from allogeneic sources, and unknown long-term effects. In 2019, the U.S. FDA issued a public safety notification stating that no EV or exosome products are approved for any indication and reporting serious adverse events in patients treated with unapproved products marketed outside investigational pathways [70]. Umbilical-cord–derived PRP, which offers a more consistent composition than autologous preparations and showed greater early pain reduction than adipose-derived MSCs in a retrospective post-arthroscopy cohort [47], faces analogous questions of donor screening, standardization, and regulatory classification. We therefore describe EV-based products as a forward-looking direction whose principal attraction—reproducible composition—is real in principle but undemonstrated in practice, and descriptions of them as “dose-definable,” “reproducible,” or the “most promising” next-generation orthobiologics are not warranted by current data.

## 8. Toward an Integrated Precision Framework—Status: A Testable Hypothesis

### 8.1. The Three Axes and Their Evidence Level

Drawing the three axes together yields a structure for organizing the literature and for designing trials (Figure 4); it is not a validated algorithm, and we present it as such. First, specify the biology: report and, where possible, select the product’s platelet dose, leukocyte and red-cell content, activation method, and generation using a shared classification (ideally the ISTH scheme), so that what is injected is known and reproducible [9,23,43]. Second, characterize the pathology: define the target tissue and its mode of failure (OA compartment and grade; tendon site and stage; symptom duration in fasciopathy; disc degeneration stage; callus deficit), because the evidence and the biological rationale differ by site [17,21,55]. Third, phenotype the patient: document structural imaging, inflammatory and metabolic status, and pain-processing and psychosocial phenotype [2,64,65,66]. Fourth, standardize and report delivery (Section 8.3). Table 6 lists the inputs of each axis together with an explicit evidence designation—established, promising, exploratory, or purely mechanistic—so that readers can see which components rest on comparative evidence and which are hypotheses. Specific product–phenotype pairings (for example, LP-PRP for “inflammatory” joints) are frequently proposed; they are not supported by comparative, biomarker-stratified trials, at least one is inconsistent with the synovial IL-6 data in Section 5.2, and none is proposed here. The expectation that aligning the three axes will reduce the heterogeneity seen in meta-analyses is a prediction of the framework, to be tested as described in Section 11.

### 8.2. Clinical Readiness: What Can Be Used Now, What Needs Validation, What Is Experimental

Because PRP, BMAC and expanded cells, and EV-based products occupy very different stages of development, and because the phenotyping tools discussed in Section 5 range from routine to research-only, presenting them within one framework risks making them appear more equivalent than they are. Table 7 therefore sorts the elements of the framework into three tiers—applicable in current practice, promising but requiring validation, and experimental—as a practical take-home for clinicians and as a statement of the evidence status of each component. Figure 5 arranges the same elements as a stepwise assessment pathway for an individual patient, with each step labelled by its readiness.

### 8.3. Image-Guided Delivery and Other Procedural Variables

Image-guided delivery is often described as the procedural substrate of precision injection, but the evidence for that role should be stated rather than assumed. It is as follows. Ultrasound guidance increases the accuracy of intra-articular injection in the hip (weighted accuracy 100% versus 72% for landmark-guided injection) [14] and of most shoulder-girdle targets—with the instructive exception of the subacromial space, where accuracy did not differ (65% versus 70%) yet guided injection nonetheless produced greater reduction in pain and improvement in function at six weeks, as it did for the biceps tendon sheath [13]. That dissociation is the point: accuracy and outcome are not the same quantity, and neither can be assumed from the other. These data establish that guidance improves placement and, at some sites, outcomes of injected drugs; they do not establish that guidance improves outcomes of orthobiologic injection specifically, which has not been tested in comparative trials, and technical accuracy does not necessarily translate into better patient-reported outcomes for every target. We therefore recommend image guidance where placement accuracy is known to be poor without it (hip, deep tendon sheaths, intradiscal and subchondral targets) and as a means of standardizing delivery in trials, while acknowledging that its incremental effect on orthobiologic efficacy is unknown. Other procedural variables—injection volume, number and interval of injections, needle fenestration or peppering, activation method, co-administration of local anesthetic (which is cytotoxic to platelets and chondrocytes in vitro), peri-procedural NSAID exposure, and the rehabilitation protocol—are rarely reported and may contribute as much to heterogeneity as product composition. That such variation is real rather than hypothetical is documented even for standardized procedures: among 92 physicians performing lumbar interlaminar epidural steroid injection, technique varied widely and only 4% reported consistently achieving bilateral contrast spread [71]. The determinants of procedural safety in image-guided interventions—real-time imaging, accurate needle placement, incremental dosing, patient selection [72]—are also the determinants of reproducible delivery. Standardized reporting of these variables is a minimum requirement for future trials (Section 11).

## 9. Regulatory and Translational Landscape

Precision matching is constrained by what regulators permit at the point of care, and classification differs materially between product classes and jurisdictions. Reviews in this field frequently summarize the landscape without primary citations and group products that are regulated differently; here each class is treated separately from primary sources, and the principal pathways are compared in Table 8. Regulatory classification is distinct from clinical endorsement and from marketing authorization: a product may be lawfully prepared and used without premarket approval and still lack evidence of efficacy.

### 9.1. United States

In the United States, human cells, tissues, and cellular and tissue-based products (HCT/Ps) are governed by 21 CFR Part 1271 [36]. A product that meets all the criteria of 21 CFR 1271.10(a)—minimal manipulation, homologous use, no combination with another article (other than water, crystalloids, or a sterilizing, preserving, or storage agent), and either no systemic effect and no dependence on the metabolic activity of living cells or, if it has such effect or dependence, autologous or closely related use—is regulated solely under Section 361 of the Public Health Service Act and does not require premarket approval; a product that fails any criterion is regulated as a drug, device, and/or biological product under Section 351 and requires an investigational new drug (IND) application and, for marketing, a biologics license [36,75]. The consequences for the products in this review are not uniform, and the common assumption that bedside PRP falls within the Section 361 category is incorrect. Autologous PRP is not an HCT/P at all: blood and blood components are excluded from the HCT/P definition under 21 CFR 1271.3(d)(2), and PRP is regulated through the medical devices used to prepare it, which are cleared via the 510(k) pathway; its use in musculoskeletal indications is at the physician’s discretion and is not an FDA-approved indication [36]. Minimally manipulated autologous bone marrow for homologous use is likewise excluded from the HCT/P definition (21 CFR 1271.3(d)(4)); FDA’s 2020 guidance on minimal manipulation and homologous use, however, states that marrow intended for a non-homologous use meets the HCT/P definition, defines homologous use by reference to the basic function of the source tissue, and identifies that function for marrow as hematopoietic (its examples of non-homologous marrow use are cardiac and neurological, not orthopedic). By that definition, BMAC injected to treat OA or a tendon would not be a homologous use and would be expected to require regulation under Section 351—an inference that the guidance does not state explicitly for orthopedic indications and that has been enforced unevenly [75]. Culture-expanded MSCs, enzymatically processed adipose-derived products, and perinatal (amniotic, umbilical-cord) products used for musculoskeletal indications are regulated as Section 351 biologics requiring an IND, and FDA has issued numerous warning letters to clinics marketing them without one [75]. For EV and exosome products, FDA stated in a 2019 public safety notification that no such products are approved for any indication and that their clinical use requires an IND [70].

### 9.2. European Union

The European Union regulates the same biology through a different structure. Cell-based products that are substantially manipulated or used non-homologously are Advanced Therapy Medicinal Products (ATMPs) under Regulation (EC) No 1394/2007, subject to centralized marketing authorization through the European Medicines Agency; the Regulation’s “hospital exemption” (Article 28, inserting Article 3(7) into Directive 2001/83/EC) permits a member state to authorize non-routine preparation of an ATMP on an individual prescription for a named patient within a hospital, under national supervision [37]. Blood-derived products such as PRP fall under the blood and blood-components framework of Directive 2002/98/EC and its implementing directives, with point-of-care autologous preparation typically handled under national blood-establishment and medical-device rules rather than as a medicinal product, and with material variation between member states [73]; that framework is being replaced by Regulation (EU) 2024/1938 on substances of human origin (SoHO), published in July 2024 and applicable from 7 August 2027, which will bring blood, tissues and cells under a single directly applicable regulation [74]. Minimally manipulated autologous bone-marrow concentrate is generally not an ATMP if it is not substantially manipulated and is used for the same essential function, but national competent authorities have reached differing conclusions on homologous use in orthopedics. EV-based products would be classified as medicinal products (biological or ATMP depending on origin and manufacture) and have no EU marketing authorization for musculoskeletal indications.

### 9.3. Implications

Two consequences follow. First, the products that are, in principle, easiest to standardize and dose—manufactured EV preparations and perinatal products—are those that require the longest regulatory path (Section 351 or ATMP), so their scientific promise is coupled to the greatest translational burden. Second, the field-wide standardization this review advocates is a regulatory as well as a scientific necessity: reproducible composition, characterization, and transparent reporting are prerequisites both for meaningful trials and for any marketing authorization that would move next-generation products from investigational into routine, reimbursable use. Classifications evolve; the summaries here reflect documents current at the time of the update search and should be confirmed against current agency guidance before application.

## 10. Feasibility, Cost, and Implementation

A framework that cannot be implemented is of limited value, and several of the tools discussed in Section 3, Section 5 and Section 7 are not available in routine practice. It is therefore useful to separate what the framework asks of a clinic from what it asks of a research program. At the clinic level, the implementable elements (Table 7, first tier) require little beyond discipline: recording the device, protocol, and, where the device reports it, the platelet and leukocyte content of the preparation; documenting the site-specific diagnosis and imaging findings; noting metabolic comorbidity and smoking; administering brief validated pain-phenotype instruments; using image guidance where accuracy is known to be poor without it; and recording procedural variables. None of these requires new equipment, and their cost is largely administrative. Point-of-care platelet counting devices are inexpensive but not universally used; complete blood counts are routine. By contrast, synovial-fluid cytokine assays, quantitative MRI sequences, multi-omic profiling, EV characterization, and machine-learning models are research infrastructure, are not reimbursed, and are unlikely to be available outside academic centers for the foreseeable future.

Cost and reimbursement are themselves barriers to precision. Orthobiologic injections are largely self-funded in most health systems; the ACR/AF, OARSI, and VA/DoD positions summarized in Table 5 are among the reasons that payers decline coverage. Adding biomarker or imaging work-up to an unreimbursed injection increases the patient’s out-of-pocket cost with, at present, no demonstrated improvement in outcome, and this is an ethical as well as an economic consideration. No cost-effectiveness analysis of phenotype-guided orthobiologic selection exists. A realistic assessment is that precision approaches may improve outcomes only marginally for some indications, may not change them for others, and may—if the neutral placebo-controlled trials generalize—reveal that certain orthobiologics have limited clinical utility regardless of stratification. Accessibility is a further concern: the centers most able to phenotype patients are the least representative of where orthobiologics are actually administered. The registry element of this agenda is at least demonstrably feasible: structured selection-and-follow-up pathways in interventional practice have produced 24-month outcome data on consecutive patients using validated instruments [76], which is what orthobiologic practice currently lacks. For these reasons, we regard the framework’s near-term value as lying chiefly in improving the design and reporting of trials and registries, and its clinical value as contingent on the results of those studies.

## 11. A Research Agenda: Designing Stratified Trials

The conclusion that stratified prospective trials are needed is shared by most reviews of this field; it is correct but insufficiently specific. Table 9 sets out the design elements we consider necessary for trials capable of testing the precision hypothesis. Three principles underlie them. First, product characterization, patient phenotyping, and delivery must be prespecified and reported; a trial that cannot describe what was injected, into whom, and how, cannot inform the framework whatever its result. Second, the decisive test of a precision approach is not efficacy within a selected subgroup but superiority of phenotype-matched allocation over conventional indication-based allocation—a design in which the matching strategy itself is randomized, or in which a prespecified biomarker–treatment interaction is the primary hypothesis. Third, trials should be powered for clinically meaningful differences against an appropriate control, should report responder proportions against a threshold validated for the instrument actually used, rather than mean differences alone—precisely because a between-group mean cannot be interpreted against an individual-level threshold (Section 4.1)— should use core outcome sets (the OMERACT–OARSI responder criteria for OA [38] and the ICON core domains for tendinopathy [39] are the available reference standards) and should follow patients for long enough to distinguish durable from transient effects and to capture subsequent interventions.

## 12. Limitations

This is a narrative review and, despite the transparency measures described in Section 2, it remains susceptible to selection and interpretation bias; it provides no pooled estimates and no formal certainty grading of its own, and the appraisal in Table 4 relies on the certainty assessments reported by the cited syntheses. Seventy-six references cannot be exhaustive across the number of indications, product classes, and cross-cutting themes addressed; we have prioritized the most recent and rigorous syntheses, guidelines, and controlled trials, and readers should be aware that several sections—notably bone healing and non-knee OA—rest on a small number of sources. The prognostic and biomarker literature is early, single-center, or extrapolated from adjacent fields such as wound healing [62], and no cited variable has been shown to modify the effect of an orthobiologic relative to a comparator. The most forward-looking data—intradiscal cell therapy, EV products, and inflammatory indices—derive from small studies that require replication [20,29,65]. Product composition and procedural reporting across the cited trials are inconsistent, which is itself the problem the review identifies. Regulatory classifications evolve and vary by jurisdiction. Two of the authors serve as Guest Editors of the Special Issue in which this article is published and were recused from its editorial handling; several authors have industry relationships disclosed below. Finally, the framework proposed here is a hypothesis: it has organized the literature coherently, but it has not been tested, and the neutral placebo-controlled trials discussed in Section 4.1, Section 4.3 and Section 4.5 are a reminder that improved stratification may not rescue interventions whose specific effect is small.

## 13. Conclusions

Orthobiologics for chronic MSK pain are neither the panacea their most enthusiastic proponents claim nor uniformly ineffective. For PRP in knee OA, the best evidence indicates a small symptomatic advantage over corticosteroid and HA, of low-to-moderate certainty and of undetermined importance to patients, which has not been reproduced against placebo in the most rigorous trial; for Achilles and lateral-elbow tendinopathy, well-controlled trials are neutral; for discogenic pain the two controlled trials disagree, one showing structural change without clinical benefit against sham and the other clinical superiority in an adjusted analysis; for cell-based and vesicle-based products more generally, the evidence is early. Part of the inconsistency across this literature plausibly reflects the use of heterogeneous products in poorly stratified populations, and the three-axis framework—product biology, tissue pathology, patient phenotype, delivered under standardized image guidance—is a coherent way to organize that heterogeneity and to design trials that could reduce it. It is, however, a hypothesis. Its elements differ widely in readiness: standardized reporting of composition and delivery, site-specific diagnosis, image-guided placement, and documentation of metabolic and pain-processing phenotype can be adopted now as good practice; biomarker-guided selection and prediction models require validation; EV-based products are experimental. Whether phenotype-matched allocation improves outcomes over conventional treatment, and for which indications, will be answered only by the stratified, adequately controlled trials outlined in Section 11—trials that must also allow for the possibility that some orthobiologics will prove to have limited clinical utility, however precisely they are matched.

## Figures and Tables

**Figure 1:**
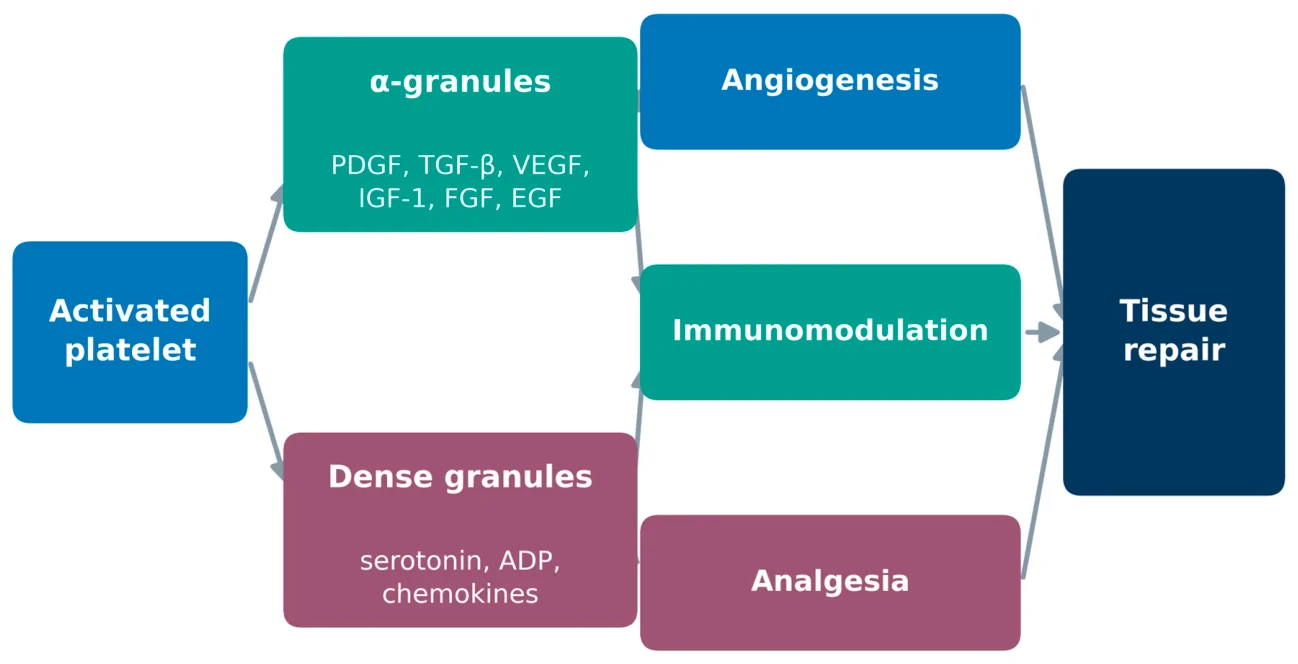
Simplified mechanistic cascade of platelet-rich plasma. Platelet activation releases α-granule growth factors (PDGF, TGF-β, VEGF, IGF-1, FGF, EGF, HGF) and dense-granule mediators (serotonin, ADP, chemokines) that drive angiogenesis, immunomodulation (M2 macrophage shift; reduced IL-1β/TNF-α), and antinociception [9,10]. The linear layout is a didactic simplification: the direction and magnitude of each effect depend on mediator dose, timing, tissue type and the local microenvironment, and convergence on “tissue repair” reflects in vitro and preclinical mechanism rather than clinically demonstrated regeneration.

**Figure 2:**
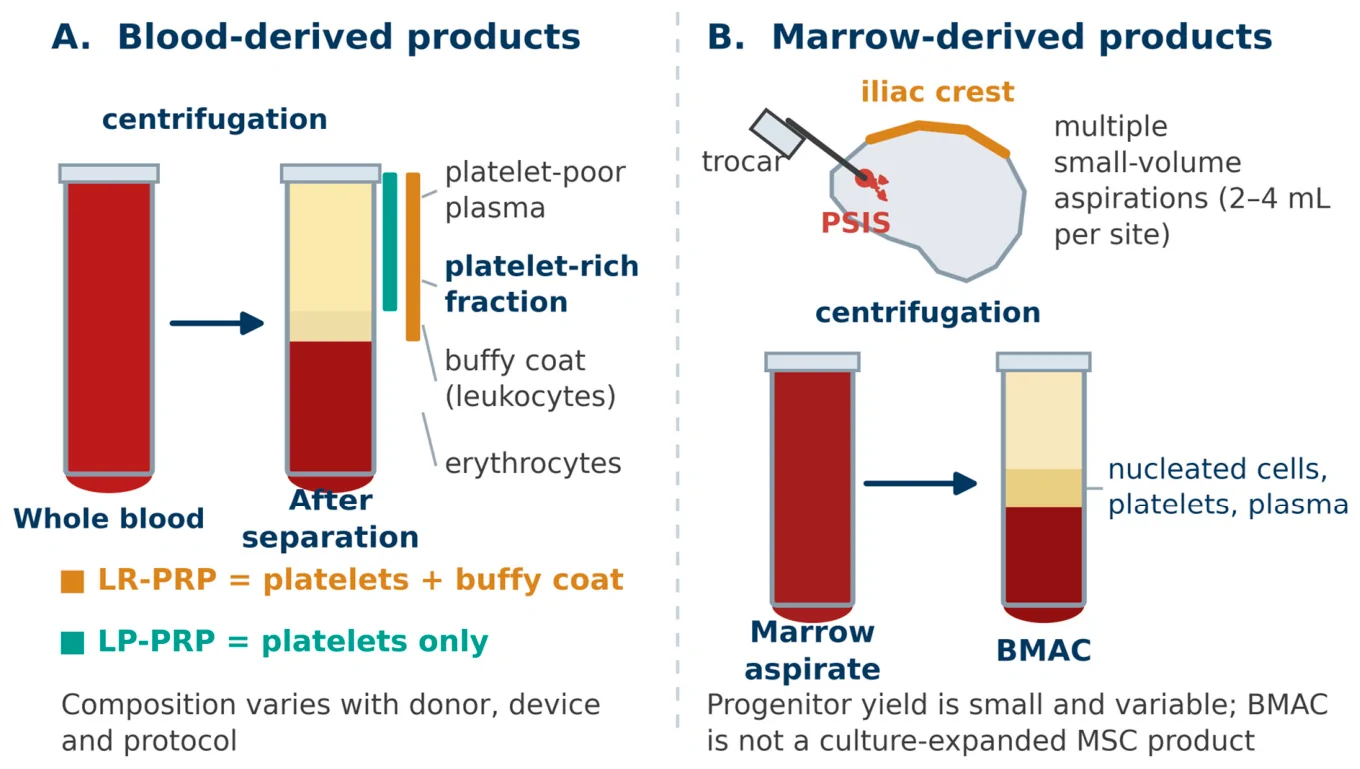
Preparation and composition of blood- and marrow-derived orthobiologics. (**A**) After centrifugation of anticoagulated whole blood, the sample separates into an erythrocyte layer, a thin buffy coat containing leukocytes and platelets, and plasma; leukocyte-poor preparations are drawn from the platelet-rich fraction immediately above the buffy coat, whereas leukocyte-rich preparations include it. Erythrocytes are excluded from intra-articular preparations because their lysis products are chondrotoxic. Composition is not fixed by the label “PRP”: in a characterization study of 335 knee-osteoarthritis patients, the mean platelet concentration reached 1.26 × 10^6^/µL (approximately 6.3-fold that of whole blood), fold-enrichment varied inversely with the baseline platelet count (6.9-fold when the baseline count was low, 4.2-fold when it was high), and the leukocyte profile shifted from neutrophil-predominant to lymphocyte-predominant [42]. (**B**) Bone marrow is aspirated from the posterior iliac crest through multiple small-volume punctures (approximately 2–4 mL per site, with the needle repositioned) to limit dilution by peripheral blood; centrifugation concentrates a buffy-coat layer (BMAC) containing nucleated cells, platelets and plasma; in the classic non-union series, iliac-crest aspirates yielded on average 612 colony-forming progenitors per cm^3^ before concentration and 2579 per cm^3^ after it, and union required more than about 1500 per cm^3^. Progenitor yield varies with the donor, the harvest site, the aspiration volume per site and the concentration protocol, and BMAC is therefore not equivalent to a defined, culture-expanded mesenchymal stromal cell product [25]. The diagram is schematic and layer proportions are not to scale. PSIS, posterior superior iliac spine; LP-PRP/LR-PRP, leukocyte-poor/leukocyte-rich platelet-rich plasma.

**Figure 4:**
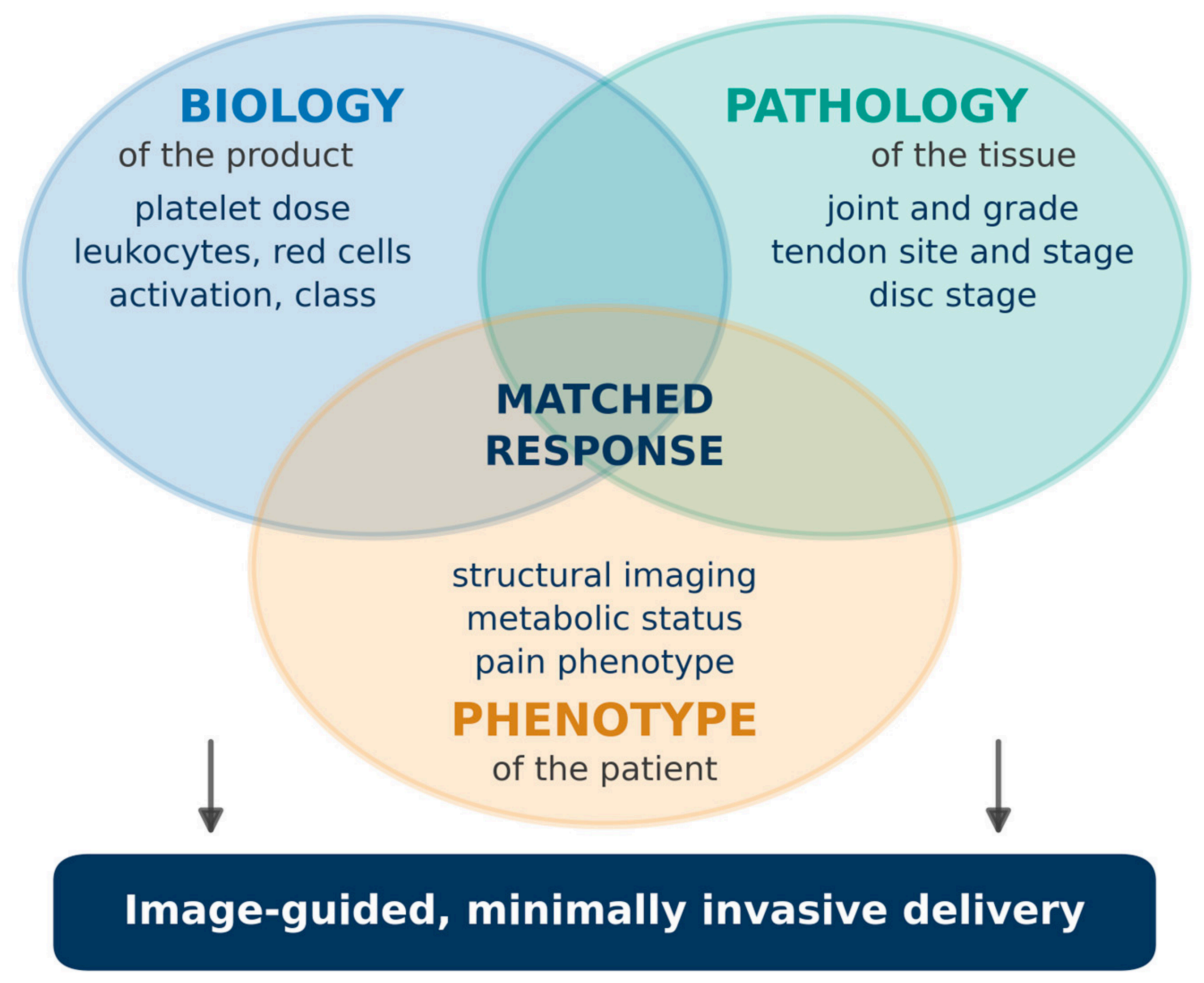
The three-axis framework as a conceptual model. An orthobiologic is hypothesized to be most likely to succeed where the biology of the product, the pathology of the target tissue, and the phenotype of the patient are jointly characterized and matched; image-guided, minimally invasive delivery—ultrasound or fluoroscopic targeting, accurate needle placement and, where recorded, real-time procedural feedback—is the procedural context in which the axes are operationalized. The model is a testable hypothesis and a structure for stratified trials, not a validated clinical algorithm; the evidence level of each input is given in Table 6 and its clinical readiness in Table 7.

**Figure 5:**
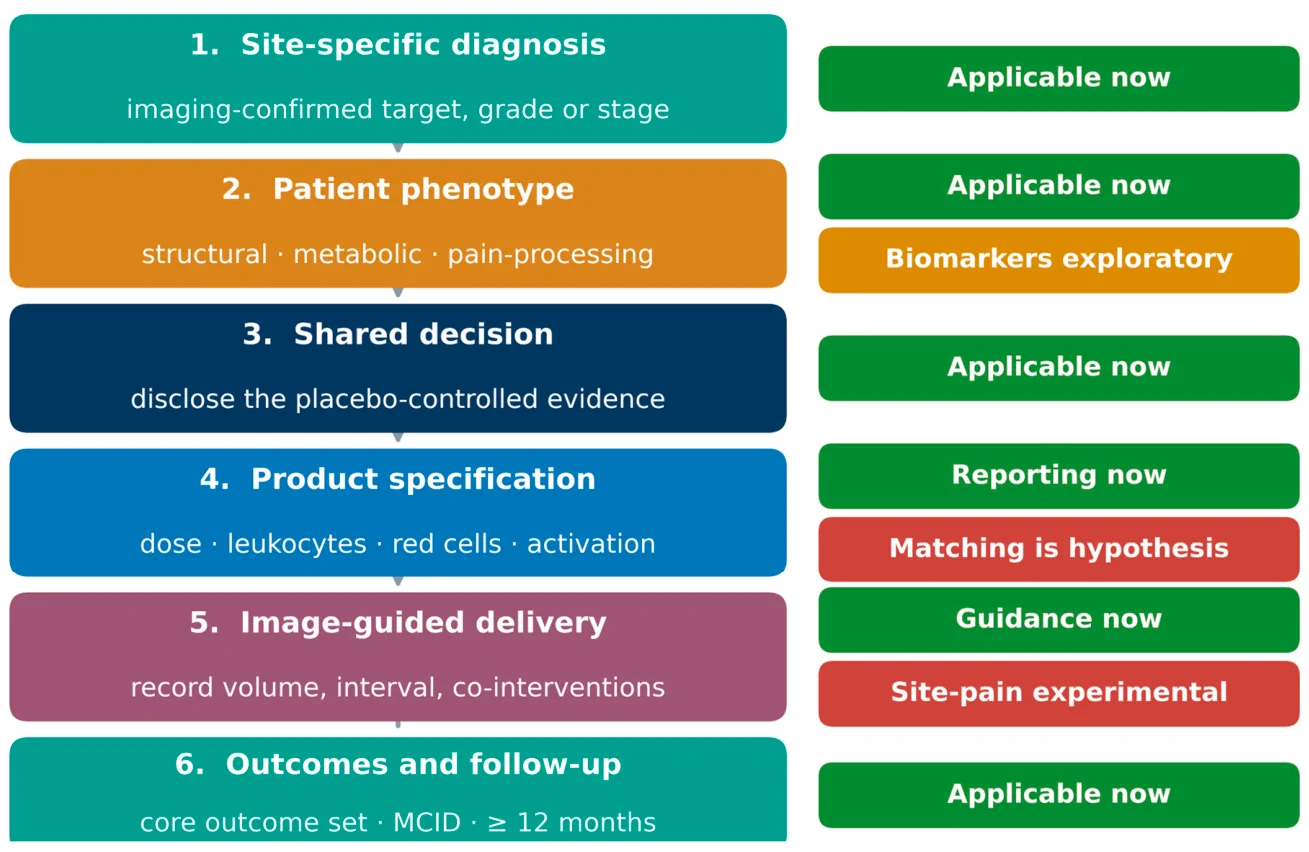
Proposed phenotype-informed assessment pathway for a patient being considered for an orthobiologic injection. Each step is labelled by its readiness: green, applicable now; amber, exploratory; red, hypothesis or experimental. Step 1 requires an imaging-confirmed target with the compartment and grade (osteoarthritis), the site and stage (tendon), the symptom duration (fascia), the degeneration stage (disc) or the nature of the osseous deficit. Step 2 documents the structural, metabolic (body-mass index, diabetes, smoking, renal function) and pain-processing or psychosocial phenotype using validated instruments, with blood-count-derived indices, synovial interleukin-6 and quantitative imaging recorded as exploratory variables. Step 3 discloses the placebo- or sham-controlled evidence for the indication, the expected benefit relative to the minimal clinically important difference, the safety profile and the cost. Step 4 records the device and protocol, the platelet dose, the leukocyte and red-cell content, the activation method and the classification used, and—for marrow products—the progenitor content; product–phenotype matching itself remains a hypothesis. Step 5 uses image guidance where accuracy is poor without it and records injection volume, number and interval, local anaesthetic, peri-procedural NSAID exposure and rehabilitation; intraprocedural pain is recorded but not used for selection. Step 6 applies a core outcome set with responder analysis anchored to the minimal clinically important difference, standardized adverse-event reporting and at least twelve months of follow-up. The pathway is offered as a structure for clinical documentation and for trial design, not as a treatment algorithm. MCID, minimal clinically important difference.

**Table 2:** Search strategy summary. The core string is abridged here; the complete strings as run in each database are in Supplementary Table S2. The third column gives the source of each specification: a cited reference where the element follows a published instrument or guideline, and the Supplementary Material where it records what the authors did.

Element	Specification	Source
Databases	PubMed/MEDLINE; Embase (Elsevier); Cochrane Central Register of Controlled Trials (CENTRAL); Web of Science Core Collection; guideline repositories (ACR/AF, OARSI, AAOS, VA/DoD, ESSKA); FDA, EMA and EUR-Lex regulatory sites	Supplementary Table S2; guideline bodies cited individually [27,32,33,34,35]
Date range	Inception to 15 January 2026 (initial search); update search 31 August 2026	Supplementary Table S2 (present review)
Core PubMed string (abridged)	(“platelet-rich plasma”[MeSH] OR PRP[tiab] OR “platelet concentrate*”[tiab] OR “bone marrow aspirate concentrate”[tiab] OR BMAC[tiab] OR orthobiologic*[tiab] OR “mesenchymal stem cells”[MeSH] OR “extracellular vesicles”[MeSH] OR exosome*[tiab]) AND (osteoarthritis[MeSH] OR tendinopathy[MeSH] OR epicondylitis[tiab] OR “rotator cuff”[tiab] OR Achilles[tiab] OR “fasciitis, plantar”[MeSH] OR fasciopathy[tiab] OR discogenic[tiab] OR “intervertebral disc degeneration”[MeSH] OR nonunion[tiab] OR “fractures, ununited”[MeSH] OR “diabetic foot”[MeSH]) AND (“precision medicine”[MeSH] OR phenotyp*[tiab] OR biomarker*[tiab] OR predict*[tiab] OR responder*[tiab] OR “machine learning”[MeSH] OR classification[tiab] OR “randomized controlled trial”[pt] OR “meta-analysis”[pt] OR guideline[pt])	Supplementary Table S2 (full strings, present review)
Language	No restriction at search; non-English records without English abstract excluded at screening	Supplementary Table S2 (present review)
Inclusion	RCTs; controlled cohorts; systematic reviews/meta-analyses/NMAs; mechanistic and characterization studies; guidelines and formal consensus; primary regulatory documents; predictor/correlate studies	Supplementary Table S2 (present review)
Exclusion	Case reports; abstracts; non-peer-reviewed sources; animal studies (except as labelled preclinical context for EVs); superseded or duplicative reviews	Supplementary Table S2 (present review)
Screening	Two independent reviewers (G.L.B., E.P.); arbitration by D.P.B.; de-duplication in EndNote	Supplementary Table S2 (present review)
Records	5967 identified → 4812 after de-duplication → 435 full texts assessed → 76 cited. Composition of the 76: 8 randomized controlled trials; 15 systematic reviews, meta-analyses or network meta-analyses; 13 observational, epidemiological or comparative clinical studies; 11 guidelines, consensus statements or reporting standards; 18 narrative or mechanistic reviews and preclinical mechanistic studies; 11 regulatory or methodological sources	Supplementary Figure S1 (present review)
Appraisal	Reported certainty (GRADE or equivalent) and risk-of-bias concerns extracted for each major evidence base; neutral/negative trials weighted equally	SANRA self-assessment in Supplementary Table S1 [40]; certainty as reported by the cited syntheses

**Table 3:** Principal platelet-derived growth factors and mediators in PRP and their biological actions.

Factor/Mediator	Principal Biological Actions	Relevance to Musculoskeletal Repair
PDGF (AA/AB/BB)	Mitogenesis and chemotaxis of fibroblasts, MSCs and smooth-muscle cells; up-regulates other growth factors; angiogenesis	Recruits and expands reparative cells; initiates tendon, ligament and bone healing [9,10]
TGF-β1	Stimulates extracellular-matrix and collagen synthesis; promotes chondrogenesis; immunomodulation (favors M2 macrophages)	Cartilage matrix production and anti-inflammatory shift in the OA joint [9,10]
VEGF	Endothelial proliferation and migration; potent pro-angiogenic signaling	Neovascularization and nutrient delivery in hypovascular tendon and bone [9,10]
IGF-1	Cell proliferation and matrix synthesis; anti-apoptotic; myogenesis	Chondrocyte and myocyte survival; synergy with PDGF/TGF-β [9,10]
bFGF (FGF-2)	Angiogenesis; fibroblast and chondrocyte proliferation; tissue remodeling	Supports granulation, tendon and cartilage cell expansion [9,10]
EGF	Epithelial and fibroblast proliferation and differentiation; supports angiogenesis	Accelerates soft-tissue and wound healing [9,10]
HGF	Anti-inflammatory and anti-fibrotic; pro-angiogenic; cytoprotective	Limits fibrosis and modulates the inflammatory microenvironment [9,10]
Serotonin, adenosine, chemokines	Bioactive amines/nucleotides and chemokines from dense granules; serotonin-mediated antinociception	Contributes to analgesia and shaping of local inflammatory tone [9,10,17]

PDGF, platelet-derived growth factor; TGF-β, transforming growth factor-beta; VEGF, vascular endothelial growth factor; IGF-1, insulin-like growth factor-1; bFGF/FGF-2, basic fibroblast growth factor; EGF, epidermal growth factor; HGF, hepatocyte growth factor. Synthesized from [9,10]. Actions are context-dependent and were characterized largely in vitro or in animal models; they do not by themselves establish clinical regeneration.

**Table 5:** Positions of major guidelines and consensus documents on platelet-rich plasma for knee osteoarthritis, verified against primary documents.

Body (Year; Scope)	Statement on PRP for Knee OA (Wording as Issued)	Strength/Level; Certainty Basis	Methodological Notes
ACR/Arthritis Foundation (2019; hand, hip, knee) [32]	“Platelet-rich plasma treatment is strongly recommended against in patients with knee and/or hip OA” (stem-cell injections likewise strongly recommended against)	Strong recommendation against	GRADE-based; systematic literature review to 2018; patient panel. The stated basis is “concern regarding the heterogeneity and lack of standardization in available preparations of platelet-rich plasma, as well as techniques used, making it difficult to identify exactly what is being injected”, not a demonstration of harm
OARSI (2019; knee, hip, polyarticular) [33]	PRP not included among recommended treatments at any level (Core, 1A, 1B, 2) for knee, hip or polyarticular OA	No recommendation issued in favour at any level of the treatment algorithm	GRADE-informed voting panel with comorbidity-specific profiles; literature to 2018
AAOS (2021, 3rd ed.; knee, nonarthroplasty) [34,58]	“Platelet-rich plasma may reduce pain and improve function in patients with symptomatic osteoarthritis of the knee”	Strength of recommendation: Limited (downgraded—higher-quality evidence lowered because of concerns in the evidence-to-decision framework)	Systematic review (2426 full texts recalled, 617 included); AAOS strength categories (strong/moderate/limited/consensus)
VA/DoD (2026; hip and knee) [35]	“There is insufficient evidence to recommend for or against platelet-rich plasma”—stated separately for knee OA and for hip OA	Neither for nor against	GRADE; U.S. veteran population; the same category was assigned in the preceding version of the guideline
ESSKA-ORBIT Part 1 (2024; knee) [27]	PRP regarded as “a valid treatment option for knee OA and as a possible first-line injectable treatment option for nonoperative management”, mainly for Kellgren–Lawrence grades 1–3	Formal consensus; of 28 statements, 3 graded A, 6 B, 7 C and 12 D—the majority rest on low-level evidence or expert opinion	RAND/Delphi-type methodology; European multidisciplinary panel; Part 2 (2025) addresses cell-based therapy [28]

Positions are reproduced from the primary documents cited; wording in quotation marks is that of the issuing body. Categories (strong against; no recommendation; limited; neither for nor against; consensus option) derive from different grading systems and are not interchangeable; in particular, “neither for nor against” denotes insufficient evidence rather than a judgement of ineffectiveness. None of the five documents recommends PRP on the basis of structural disease modification.

**Table 6:** The three-axis precision framework with an evidence designation for each input.

Axis	Key Question	Inputs	Evidence Designation for Use in Treatment Selection
Biology (product)	What exactly is injected, and is it reproducible?	Platelet dose; leukocyte-rich vs. -poor; RBC content; activation; generation (PRP vs. L-PRF); ISTH/other classification; for marrow products, progenitor content [9,23,25,42,43]	Reporting: established as good practice. Selecting composition for a given pathology: exploratory (no comparative trial shows that composition-matched selection improves outcome)
Pathology (target)	Does the biology fit the tissue’s mode of failure, and what is the best comparative evidence at this site?	OA compartment/grade; tendon site and stage; fasciopathy symptom duration; disc stage; type of osseous deficit [17,18,20,21,55]	Site-specific diagnosis: established. Site-specific product choice: promising for plantar fasciopathy (time-course data); exploratory elsewhere; neutral for Achilles/lateral elbow and disc
Phenotype (patient)	Which patient is likely to benefit, relative to alternatives?	Structural MRI; synovial IL-6; CBC-derived indices; metabolic comorbidity; pain-processing and psychosocial measures; ML risk scores [2,62,63,64,65]	Metabolic and pain-phenotype documentation: established as prognostic. Inflammatory biomarkers: exploratory. ML models: exploratory (single-center, unvalidated). No input is a validated effect modifier
Delivery (procedure)	Was the intended compartment reached, and how was the injection performed?	Image guidance; target compartment; volume; number/interval; local anesthetic; NSAID exposure; rehabilitation [13,14]	Image guidance for accuracy: established for several joints; effect on orthobiologic outcomes: untested. Other procedural variables: exploratory; should be reported

Designations: established—supported by comparative evidence or accepted good practice; promising—consistent signals requiring confirmation; exploratory—single studies, uncontrolled data, or mechanistic rationale only; mechanistic—in vitro/preclinical rationale without human data. RBC, red blood cell; CBC, complete blood count; ML, machine learning.

**Table 7:** Clinical readiness of the elements of the precision-orthobiologic framework.

Tier	Elements	Basis
Applicable in current clinical practice	Documentation and reporting of PRP composition (platelet dose, leukocyte/RBC content, activation, device); imaging-confirmed, site-specific diagnosis; image-guided delivery where accuracy is known to matter; assessment and documentation of metabolic comorbidity, smoking, and pain-processing/psychosocial phenotype; discussion of realistic expected benefit and of neutral trial evidence; preference for autologous blood-derived products over cell-based products on safety and evidence grounds	Good practice, established prognostic factors, guideline-consistent; does not by itself establish that outcomes improve [13,14,23,42,64,66]
Promising but requiring validation	Site- and time-specific product choice in plantar fasciopathy; CBC-derived inflammatory indices; synovial cytokine profiling; quantitative imaging phenotypes; externally validated prediction models; standardized BMAC progenitor quantification	Consistent signals from cohorts, single trials, or subgroup analyses; no demonstrated treatment-by-biomarker interaction [17,18,19,55,63,65]
Experimental/emerging	Platelet- and MSC-derived EV products; perinatal allogeneic PRP; intradiscal cell therapy in selected phenotypes; pathological-site pain as a targeting signal; multi-omic stratification; ML-directed treatment allocation	Preclinical or first-in-human data only; unresolved manufacturing, dosing, regulatory, and safety questions [26,29,45,47,56,67,68]

**Table 8:** Regulatory pathways for orthobiologic product classes in the United States and the European Union.

Product Class	United States (FDA)	European Union (EMA/National Authorities)
Autologous PRP (point of care)	Not an HCT/P (blood component exclusion, 21 CFR 1271.3(d)(2)); preparation devices cleared via 510(k); MSK use at physician discretion, not an approved indication [36]	Blood component under Directive 2002/98/EC (SoHO Regulation (EU) 2024/1938 from August 2027); point-of-care autologous use governed by national blood-establishment/device rules; not a medicinal product in most member states [73,74]
Autologous BMAC (point of care)	Minimally manipulated marrow for homologous use excluded from HCT/P (21 CFR 1271.3(d)(4)); orthopedic use is non-homologous under the 2020 guidance’s definition (basic function of marrow = hematopoietic) → Section 351 expected by inference; uneven enforcement [75]	Generally not an ATMP if not substantially manipulated and same essential function; national authorities differ on homologous use in orthopedics [37]
Culture-expanded MSCs; adipose-derived (enzymatic) products	Section 351 biologic; IND required; BLA for marketing [36,75]	ATMP (Regulation (EC) 1394/2007); centralized authorization; hospital exemption for non-routine named-patient use [37]
Perinatal (amniotic/umbilical) products for MSK use	Section 351 biologic; non-homologous for MSK indications per 2020 guidance; IND required; warning letters issued [75]	ATMP or biological medicinal product depending on manipulation; no MSK marketing authorization [37]
EV/exosome products	No approved products; IND required; 2019 public safety notification [70]	Medicinal product (biological/ATMP); no marketing authorization for MSK indications

HCT/P, human cells, tissues, and cellular and tissue-based products; IND, investigational new drug; BLA, biologics license application; ATMP, advanced therapy medicinal product; MSK, musculoskeletal. Summaries reflect primary documents current at 31 August 2026 and are not legal advice.

**Table 9:** Recommended design elements for future trials of orthobiologics intended to test a precision (phenotype-matched) hypothesis.

Domain	Minimum Requirement	Preferred
Product characterization	Report device/protocol, platelet concentration and dose, leukocyte and RBC content, activation, volume, storage time; use a recognized classification (e.g., ISTH) [23]	Batch-level assay of platelet count and key growth factors; for marrow products, CFU-F/progenitor quantification [25]; for EVs, MISEV2023-compliant characterization [26]
Patient phenotyping (prespecified)	Site-specific diagnosis with imaging; KL grade or equivalent; BMI, diabetes, smoking; validated pain-phenotype measures (e.g., Central Sensitization Inventory, painDETECT, PROMIS mental/physical) [64,65]	Synovial or peripheral inflammatory biomarkers with prespecified cut-offs; quantitative imaging; biobanking for later analysis
Biomarker collection standards	Standardized timing (pre-injection), handling, and assay platforms; pre-registered analysis plan for interactions	Central laboratory; blinded assessment; external validation cohort [30,63,65]
Design	Randomized; blinded participant and assessor; placebo/sham or active comparator justified by the question; allocation concealment; prespecified subgroup/interaction analyses with adequate power	Randomization of the matching strategy itself (phenotype-matched vs. conventional allocation), or adaptive enrichment designs [7,20,30]
Delivery	Image-guided with documented target; standardized volume, number, and interval; recorded local anesthetic and NSAID use; standardized rehabilitation [13]	Confirmation of intra-compartmental placement (contrast/ultrasound); procedural video or image archive
Outcomes	Core outcome set for the indication (OMERACT–OARSI responder criteria [38]; ICON core domains for tendinopathy [39]); MCID-anchored responder analysis [31]; pain, function, quality of life; adverse events with standardized definitions	Objective structural/functional endpoints as secondary; health economics; subsequent interventions
Follow-up	Minimum 12 months for OA, tendon, and disc; 6 months for fasciopathy with 12-month secondary	24 months or longer for durability and disease modification [7,28,56]
Reporting and registration	Prospective registration; CONSORT; full reporting of composition and delivery; data sharing	Individual-participant data available for meta-analysis [23,26,40]

## Data Availability

No new data were created or analyzed in this study. Data sharing is not applicable to this article.

## Supplementary Materials

The following supporting information can be downloaded at: https://www.mdpi.com/article/10.3390/medicina62101881/s1, Table S1: SANRA (Scale for the Assessment of Narrative Review Articles) self-assessment; Table S2: Full search strategies; Figure S1: Literature identification and selection flow.

## Abbreviations

AAOS	American Academy of Orthopaedic Surgeons
ACR	American College of Rheumatology
ADAMTS5	a disintegrin and metalloproteinase with thrombospondin motifs 5
AOFAS	American Orthopaedic Foot and Ankle Society
ATMP	advanced therapy medicinal product
AUC	area under the curve
BLA	biologics license application
BM-MSC	bone marrow–derived mesenchymal stromal cell
BMAC	bone marrow aspirate concentrate
CBC	complete blood count
CFR	Code of Federal Regulations
CFU-F	colony-forming unit–fibroblast
EMA	European Medicines Agency
ESSKA	European Society of Sports Traumatology, Knee Surgery and Arthroscopy
EV/PDEV	extracellular vesicle/platelet-derived extracellular vesicle
FDA	U.S. Food and Drug Administration
GRADE	Grading of Recommendations Assessment, Development and Evaluation
HA	hyaluronic acid
HCT/P	human cells, tissues, and cellular and tissue-based products
HHS	Harris Hip Score
ICON	International Scientific Tendinopathy Symposium Consensus
IL-6/IL-1β	interleukin-6/interleukin-1 beta
IND	investigational new drug
ISTH	International Society on Thrombosis and Haemostasis
KL	Kellgren–Lawrence
KOOS	Knee injury and Osteoarthritis Outcome Score
LP-PRP/LR-PRP	leukocyte-poor/leukocyte-rich platelet-rich plasma
MCID	minimal clinically important difference
MISEV	Minimal Information for Studies of Extracellular Vesicles
ML	machine learning
MPC	mesenchymal precursor cell
MRI	magnetic resonance imaging
MSC	mesenchymal stromal cell
MSK	musculoskeletal
NLR/PLR/SII	neutrophil-to-lymphocyte ratio/platelet-to-lymphocyte ratio/systemic immune-inflammation index
NMA	network meta-analysis
NSAID	non-steroidal anti-inflammatory drug
OA	osteoarthritis
OARSI	Osteoarthritis Research Society International
OMERACT	Outcome Measures in Rheumatology
PROMIS	Patient-Reported Outcomes Measurement Information System
PRP	platelet-rich plasma
PSIS	posterior superior iliac spine
RBC	red blood cell
RCT	randomized controlled trial
RoB	risk of bias
SANRA	Scale for the Assessment of Narrative Review Articles
SCS	spinal cord stimulation
VA/DoD	U.S. Department of Veterans Affairs/Department of Defense
VAS	visual analog scale
VISA-A	Victorian Institute of Sport Assessment–Achilles
WOMAC	Western Ontario and McMaster Universities Osteoarthritis Index
