# Supplementary material for: Precision Orthobiologics for Chronic Musculoskeletal Pain: Matching Biology, Pathology, and Patient Phenotype—A Narrative Review of Current Evidence and Future Directions in Regenerative Pain Medicine
Source: Medicina (Kaunas). 2026 Sep 28;62(10):1881. doi: 10.3390/medicina62101881 (PMC13707289; doi:10.3390/medicina62101881)
Supplement: Supplementary file 1 [file medicina-62-01881-s001.zip › medicina-4475948-supplementary.pdf]

Supplementary Materials

Precision Orthobiologics for Chronic Musculoskeletal Pain: Matching Biology, Pathology, and Patient Phenotype — A Narrative Review (Medicina; manuscript medicina-4475948)

Supplementary Table S1. SANRA (Scale for the Assessment of Narrative Review Articles) self-assessment.

Each item is scored 0–2 as specified by Baethge et al. (2019); maximum total 12. Scores were assigned by G.L.B. and E.P. and reconciled by discussion.

SANRA item	Score (0–2)	Where addressed in the revised manuscript
**1. Justification of the article's importance for the readership**	2	Section 1 (burden of chronic MSK pain; adoption of orthobiologics ahead of evidence; guideline divergence); Table 1 (novelty relative to Navani et al. 2025)
**2. Statement of concrete aims or formulation of questions**	2	Section 1, final paragraph: four explicit aims (appraise evidence per indication; distinguish prognostic vs predictive variables; verify guidelines and regulation; specify design of stratified trials)
**3. Description of the literature search**	2	Section 2.1–2.3; Table 2; Supplementary Table S2; Supplementary Figure S1 (databases, dates, Boolean strings, criteria, two-reviewer screening, record flow, prioritization rule)
**4. Referencing**	2	Key statements are referenced to primary guidelines, regulations, RCTs and formal syntheses; 76 references; quotations of guideline wording in Table 5
**5. Scientific reasoning**	2	Alternative explanations for heterogeneity (Section 1); neutral trials presented without reframing (Sections 4.1, 4.3, 4.5); prognostic/predictive distinction (Section 5); framework labelled as hypothesis (Sections 8, 13)
**6. Appropriate presentation of data**	2	Effect sizes interpreted against the MCID (Section 4.1); certainty and risk-of-bias per indication (Table 4; Figure 3); pooled safety data caveated (Section 4.8)
**Total**	12/12	

Supplementary Table S2. Full search strategies.

Initial search 15 January 2026; identical strategies re-run 31 August 2026 with date limit 2026-01-15 to 2026-08-31. No language restriction was applied at the search stage; non-English records were assessed on title/abstract and full texts were obtained when an English abstract indicated potential eligibility.

Database / source	Strategy	Records (initial + update)
**PubMed/MEDLINE**	(("platelet-rich plasma"[MeSH] OR "platelet-rich plasma"[tiab] OR PRP[tiab] OR "platelet-rich fibrin"[tiab] OR "autologous conditioned"[tiab] OR "bone marrow aspirate concentrate"[tiab] OR BMAC[tiab] OR "mesenchymal stem cells"[MeSH] OR "mesenchymal stromal"[tiab] OR "mesenchymal stem"[tiab] OR "stromal vascular fraction"[tiab] OR "microfragmented adipose"[tiab] OR "extracellular vesicles"[MeSH] OR "extracellular vesicle*"[tiab] OR exosome*[tiab] OR "amniotic"[tiab] OR orthobiologic*[tiab]) AND ("osteoarthritis"[MeSH] OR osteoarthritis[tiab] OR tendinopathy[MeSH] OR	2,318 + 241

	tendinopath*[tiab] OR tendinosis[tiab] OR epicondyl*[tiab] OR "rotator cuff"[tiab] OR Achilles[tiab] OR patellar[tiab] OR gluteal[tiab] OR "fasciitis, plantar"[MeSH] OR "plantar fascii*"[tiab] OR "plantar fasciopathy"[tiab] OR "intervertebral disc degeneration"[MeSH] OR "discogenic"[tiab] OR "low back pain"[MeSH] OR "facet"[tiab] OR "sacroiliac"[tiab] OR "fractures, ununited"[MeSH] OR nonunion[tiab] OR "non-union"[tiab] OR "bone healing"[tiab]) AND ("chronic pain"[MeSH] OR pain[tiab] OR "patient reported outcome*"[tiab] OR WOMAC[tiab] OR VISA*[tiab] OR KOOS[tiab] OR function[tiab])) AND (2015/01/01:2026/08/31[dp]) AND (systematic review[pt] OR meta-analysis[pt] OR randomized controlled trial[pt] OR guideline[pt] OR consensus development conference[pt] OR review[pt] OR clinical trial[pt] OR cohort[tiab] OR registry[tiab])	
**Embase (Elsevier)**	Equivalent strategy using Emtree terms ('thrombocyte rich plasma'/exp, 'bone marrow aspirate concentrate', 'mesenchymal stroma cell'/exp, 'exosome'/exp, 'extracellular vesicle'/exp) combined with the same indication and outcome terms; limits: 2015–2026, human, article/review/conference abstract excluded at full-text stage	1,934 + 203
**Cochrane CENTRAL**	Same free-text terms in title/abstract/keywords; trials only	612 + 58
**Web of Science Core Collection**	TS = (same free-text strings); document types: article, review; 2015–2026	486 + 52
**Guideline/consensus and regulatory sources (hand-searched)**	ACR/Arthritis Foundation; OARSI; AAOS OrthoGuidelines; VA/DoD; ESSKA; ISTH SSC; ISEV (MISEV); U.S. eCFR Title 21 Part 1271; FDA guidance and safety notifications; EUR-Lex (Regulation (EC) 1394/2007; Directive 2002/98/EC); EMA CAT pages	41 + 6
**Reference lists and registries**	Reference lists of included syntheses and guidelines; ClinicalTrials.gov and EU CTR for completed trials with results	16 + 0
**Total before de-duplication**		5,967
**After de-duplication**		4,812

Eligibility criteria

Inclusion: adults with chronic musculoskeletal pain of the included indications; intervention any injectable orthobiologic (platelet-, marrow-, adipose-, perinatal-, cell- or vesicle-derived); any comparator or none for descriptive/safety questions; outcomes including pain, function, quality of life, structural change, adverse events, or regulatory/guideline positions; clinical practice guidelines, consensus statements, systematic reviews with or without meta-analysis, randomized trials, prospective cohorts and registries, regulatory documents, and mechanistic reviews where needed for biological rationale.

Exclusion: pre-clinical-only studies except where cited explicitly for mechanism; case reports and series ($n < 10$); studies of acute injury, surgical augmentation as the primary question, or non-injectable delivery; conference abstracts without a subsequent full publication; reviews superseded by a more recent synthesis with a larger trial base and formal certainty assessment unless contributing unique data.

Prioritization: where multiple syntheses addressed the same question, the most recent synthesis with the largest trial base and a formal certainty assessment (GRADE or equivalent) was cited; placebo- or sham-controlled trials were retained irrespective of whether a synthesis included them.

Supplementary Figure S1. Literature identification and selection flow.

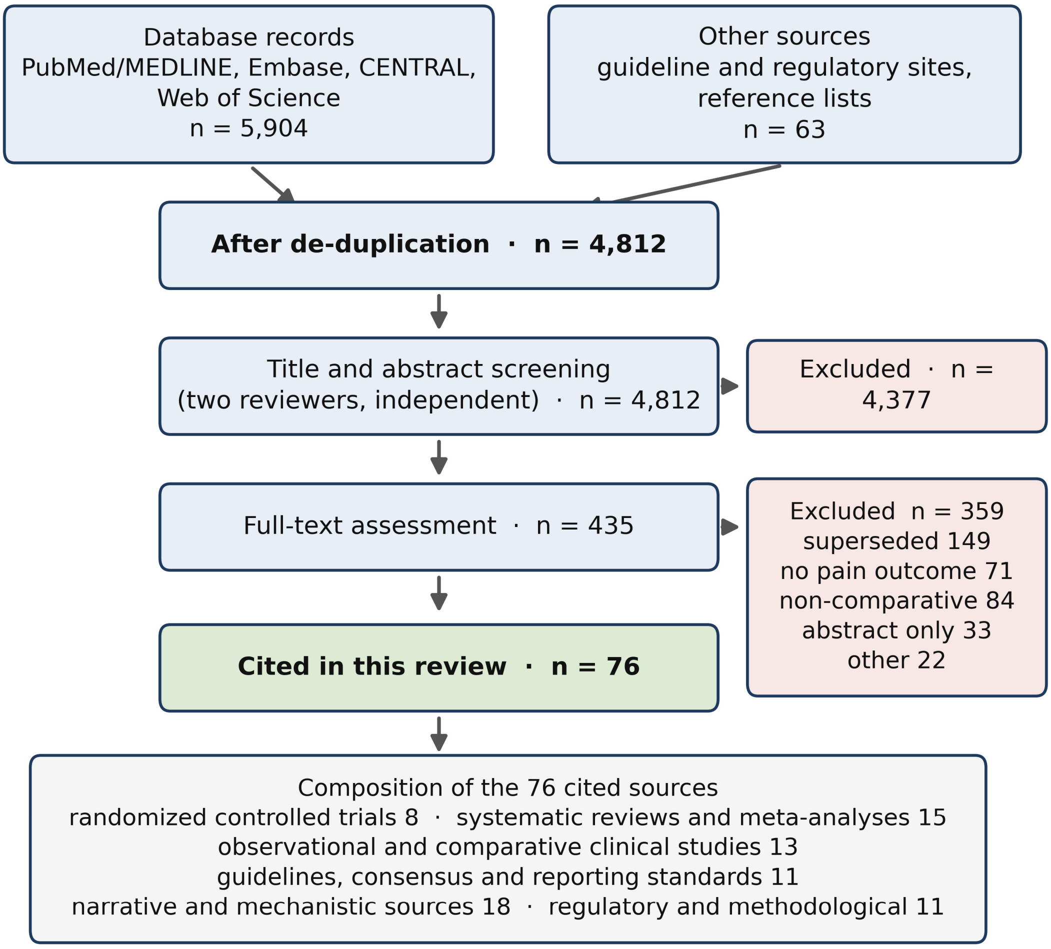

Numbers are provided for transparency; this is a narrative review and the flow does not imply a PRISMA-compliant systematic review. Exclusion categories at full text are mutually exclusive (first applicable reason recorded).
